# Genome-wide association study of post COVID-19 syndrome in a population-based cohort in Germany

**DOI:** 10.1038/s41598-025-00945-z

**Published:** 2025-05-06

**Authors:** Anne-Kathrin Ruß, Stefan Schreiber, Wolfgang Lieb, J. Janne Vehreschild, Peter U. Heuschmann, Thomas Illig, Katharina S. Appel, Maria J. G. T. Vehreschild, Dagmar Krefting, Lennart Reinke, Alin Viebke, Susanne Poick, Stefan Störk, Jens-Peter Reese, Thomas Zoller, Lilian Krist, David Ellinghaus, Bärbel U. Foesel, Christian Gieger, Bettina Lorenz-Depiereux, Martin Witzenrath, Gabriele Anton, Michael Krawczak, Jan Heyckendorf, Thomas Bahmer

**Affiliations:** 1 Institute of Medical Informatics and Statistics, University Medical Center Schleswig-Holstein, Kiel University, Brunswiker Straße 10, 24113 Kiel, Germany; 2 Institute of Epidemiology, University Medical Center Schleswig-Holstein, Kiel University, Kiel, Germany; 3 Department of Internal Medicine I, University Medical Center Schleswig-Holstein, Kiel, Germany; 4 Institute of Digital Medicine and Clinical Data Science, Faculty of Medicine, Goethe University Frankfurt, Frankfurt, Germany; 5 Department I of Internal Medicine, Faculty of Medicine, University Hospital CologneUniversity of Cologne, Cologne, Germany; 6 Institute of Clinical Epidemiology and Biometry, University of Würzburg, Würzburg, Germany; 7 Institute of Medical Data Science, University Hospital Würzburg, Würzburg, Germany; 8 Clinical Trial Center, University Hospital Würzburg, Würzburg, Germany; 9 Hannover Unified Biobank, Hannover Medical School, Hannover, Germany; 10 Biomedical Research in Endstage and Obstructive Lung Disease Hannover, German Center for Lung Research, Hannover, Germany; 11 Medical Department 2, Center for Internal Medicine, University Hospital Frankfurt, Goethe University Frankfurt, Frankfurt, Germany; 12 Department of Medical Informatics, University Medical Center Göttingen, Göttingen, Germany; 13 Campus Institute Data Sciences, Göttingen, Germany; 14 Department of Clinical Research and Epidemiology, Comprehensive Heart Failure Center, University Hospital Würzburg, Würzburg, Germany; 15 Department of Internal Medicine I, University Hospital Würzburg, Würzburg, Germany; 16 Faculty of Health Sciences, THM University of Applied Sciences, Gießen, Germany; 17 Department of Infectious Diseases, Respiratory and Critical Care Medicine, Charité - Universitätsmedizin Berlin, Freie Universität Berlin, Humboldt-Universität zu Berlin, Berlin, Germany; 18 Institute of Social Medicine, Epidemiology and Health Economics, Charité - Universitätsmedizin Berlin, Freie Universität Berlin, Humboldt-Universität zu Berlin, Berlin, Germany; 19 Institute of Clinical Molecular Biology, University Medical Center Schleswig-Holstein, Kiel University, Kiel, Germany; 20 Institute of Epidemiology, Research Unit of Molecular Epidemiology, Helmholtz Munich - German Research Center for Environmental Health, Neuherberg, Germany; 21 Department of Infectious Diseases and Critical Care Medicine, Charité - Universitätsmedizin Berlin, Freie Universität Berlin, Humboldt-Universität zu Berlin, Berlin, Germany; 22 CAPNETZ Stiftung, Hannover, Germany; 23 Medical School OWL, Bielefeld University, Bielefeld, Germany; 24 Airway Research Center North (ARCN), German Center for Lung Research (DZL), Großhansdorf, Germany; 25 Leibniz Lung Clinic, Kiel, Germany

**Keywords:** SARS-CoV-2, Long-COVID, Genotype-phenotype association, Linkage disequilibrium, Single nucleotide polymorphism, Olfactory receptor, Virus repression, Macrophage activation, Genetic association study, Infectious diseases, Risk factors, Genetics research

## Abstract

If health impairments due to coronavirus disease 2019 (COVID-19) persist for 12 weeks or longer, patients are diagnosed with Post-COVID Syndrome (PCS), or Long-COVID. Although the COVID-19 pandemic has largely subsided in 2024, PCS is still a major health burden worldwide, and identifying potential genetic modifiers of PCS remains of great clinical and scientific interest. We therefore performed a case-control type genome-wide association study (GWAS) of three recently developed PCS (severity) scores in 2,247 participants of COVIDOM, a prospective, multi-centre, population-based cohort study of SARS-CoV-2-infected individuals in Germany. Each PCS score originally represented the weighted sum of the binary indicators of all, or a subset, of 12 PCS symptom complexes, assessed six months or later after the PCR test-confirmed SARS-CoV-2 infection of a participant. For various methodical reasons, however, the PCS scores were dichotomized along their respective median values in the present study, prior to the GWAS. Of the 6,383,167 single nucleotide polymorphisms included, various variants were found to be associated with at least one of the PCS scores, although not at the stringent genome-wide statistical significance level of 5 × 10^− 8^. With *p* = 6.6 × 10^− 8^, however, the genotype-phenotype association of SNP rs9792535 at position chr9:127,166,653 narrowly missed this threshold. The SNP is located in a region including the *NEK6*, *PSMB7* and *ADGRD2* genes which, however, does not immediately suggest an etiological connection to PCS. As regards functional plausibility, variants of a possible effect mapped to the olfactory receptor gene region (lead SNP rs10893121 at position chr11:123,854,744; *p* = 2.5 × 10^− 6^). Impairment of smell and taste is a pathognomonic feature of both, acute COVID-19 and PCS, and our results suggest that this connection may have a genetic basis. Three other genotype-phenotype associations pointed towards a possible etiological role in PCS of cellular virus repression (*CHD6* gene region), activation of macrophages (*SLC7A2*) and the release of virus particles from infected cells (*ARHGAP44*). All other gene regions highlighted by our GWAS did not relate to pathophysiological processes currently discussed for PCS. Therefore, and because the genotype-phenotype associations observed in our GWAS were generally not very strong, the complexity of the genetic background of PCS appears to be as high as that of most other multifactorial traits in humans.

**Supplementary Information:**

The online version contains supplementary material available at 10.1038/s41598-025-00945-z.

## Introduction

The global pandemic of coronavirus disease 2019 (COVID-19), a potentially fatal condition caused by infection with the newly identified coronavirus SARS-CoV-2, started in China in late 2019. While respiratory symptoms such as cough and dyspnoea have since been found to predominate in COVID-19 patients, the disease also affects multiple other organs to a varying degree^1,2^. Severe cases might ultimately suffer from acute respiratory distress syndrome with a potentially fatal course. If COVID-19-associated health impairments persist until 12 weeks or later after the initial infection, and if other causes can be ruled out, patients are diagnosed with Post-COVID Syndrome (PCS), or Long-COVID^3^ (https://www.nice.org.uk/Guidance/NG188, accessed 20 March 2025). Although the COVID-19 pandemic has lost most of its immediate threat in 2025, PCS still poses a significant public health burden worldwide because recovery from COVID-19 is often slow and the risk of chronic manifestation of symptoms is high.

The course of COVID-19 is a prominent example of the human response to viral infections, which results from the complex interaction of a variety of internal and external factors. In addition to viral properties and environmental conditions, the physiological characteristics of the host contribute to this coping process as well. While the innate and adaptive immune systems clearly play a central role in this regard, the general physical condition and the psychological resilience of the affected individual are also likely to determine whether, and how well, the infection is overcome^4^. Notably, as with most complex human traits, differences regarding such host factors can be assumed to be influenced, at least in part, by genetic variation.

Over the past 25 years, most research into the genetic basis of complex human traits has been conducted in the form of case-control studies that exploit the statistical association, at the population level, between genetic variants that are truly causative of the trait of interest and the genotypes of, usually functionless, single nucleotide polymorphisms (SNPs). These so-called ‘genome-wide association studies’ (GWAS) have led to the identification of genotypic associations with over 5,000 human traits, according to the European Bioinformatics Institute GWAS catalogue (http://www.ebi.ac.uk/gwas, accessed 20 March 2025).

While a considerable number of GWAS were performed for acute COVID-19 shortly after the outbreak of the pandemic^5–7^, only two such studies have been published for PCS to date. The study by Lammi et al.^8^, which relied upon resources from the COVID-19 Host Genetics Initiative, comprised > 6,000 cases and > 1 million population controls from 24 studies in Europe, North America and East Asia. Through meta-analysis, a single genome-wide significant association with PCS was identified, namely of the *FOXP4* locus that had been connected before to acute COVID-19 severity as well. Although acute disease severity is a known risk factor for PCS^4^, the authors of the GWAS emphasized that this connection was not sufficient to explain the association between PCS and *FOXP4* genetic variation. The second GWAS, by Taylor et al.^9^, was conducted in two sub-cohorts of the Sano Genetics Long-COVID GOLD study, where > 90% participants had self-reported “white” ethnicity. Taking a combinatorial approach that allowed for gene-gene interactions, the study identified 73 genes to be associated with PCS severity in at least one of the two sub-cohorts. Notably, nine of these genes had also been connected to acute COVID-19 severity before.

The power of a GWAS and the validity of its results critically depend upon the definition of the underlying phenotype. A score developed by our group in 2022 specifically for the purpose of phenotyping PCS patients allows their overall COVID-19-related health problems to be severity-graded on the basis of 12 self-assessed symptom complexes^4^. This PCS score was also shown to have two main predictors, namely acute COVID-19 severity and individual resilience, both of which are rather differently associated with the 12 symptom complexes underlying the score^4^. This specificity of association inspired the definition of two additional PCS scores that encompass only subsets of the 12 symptom complexes, and that allow for more accurate assessment of PCS severity in the context of both research and clinical care^10^.

Despite the subsiding of the pandemic, identifying genetic modifiers of the long-term consequences of COVID-19 still remains of great practical and scientific interest, not least with a view to the development of efficient clinical treatment and patient management strategies. Therefore, we performed a GWAS of the three abovementioned PCS scores in participants of COVIDOM, a prospective, multi-centre, population-based cohort study of SARS-CoV-2-infected individuals in Germany.

## Materials and methods

### The COVIDOM study

The COVIDOM study of the long-term health effects of COVID-19 started in Germany in October 2020 and was funded by the German Federal Ministry of Education and Research as part of the National Pandemic Cohort Network (NAPKON, grant number: 01KX2121). Participants of this prospective, multi-centre, population-based cohort study were recruited six months or later after their PCR test-confirmed SARS-CoV-2 infection. Between November 2020 and May 2023, a total of 3,632 probands took part in an online survey and were subsequently examined during their visit at one of the COVIDOM study sites in Kiel (*n* = 2,557), Berlin (*n* = 469) and Würzburg (*n* = 606). For further details about the COVIDOM study, see Horn et al.^11^ and Bahmer et al.^4^.

#### Study protocol

Data acquisition in COVIDOM took place in two phases. First, a questionnaire-based online interview was carried out to collect relevant socio-demographic data of the participants as well as self-reported information about the acute phase of their infection. During the study site visit, every participant underwent examinations covering various medical areas, including anthropometry, pulmonology, olfactory and gustatory functions, cardiology, neurology, geriatrics and hepatology. Validated tools such as the PHQ-8 and FACIT-F questionnaires were used for additional data acquisition. These procedures are currently repeated annually as part of a long-term follow-up of COVIDOM participants.

### Phenotype definition

The PCS score developed by Bahmer et al.^4^ differentially weighs the presence of 12 clinical symptom complexes to quantify the severity of the COVID-19-related long-term health problems of patients. In the present GWAS, a median split according to the PCS score served to divide COVIDOM participants into two PCS severity groups. The same approach was taken for two recently proposed PCS sub-scores that are more specific than the original score for the two main risk factors for severe PCS^10^, namely low individual resilience and severe acute illness. Both risk factors were themselves considered as outcome variables in additional GWAS after dichotomization along the median value of the BRS Brief Resilience Scale^12^, or employing a threshold of four for the number of self-reported severe/life threatening acute phase symptoms of COVID-19.

While the dichotomization of quantitative measurements is a popular means in medical research to reduce the complexity of the analysis and interpretation of study data, it is also known to reduce statistical power and to potentially introduce bias^13^. However, these drawbacks primarily concern continuous measurements with ‘well-behaved’ statistical distributions, mostly normal or log-normal, for which the highly implemented, usually model-based analysis methods available to researchers are known to be valid. For measurements with more irregular properties such as strong skewness, multi-modality or non-linear relations to predictors and covariates, in contrast, dichotomization may be justified in order to balance power loss and methodological validity, i.e. correctness of p values^14^. The three PCS scores studied here definitely belong to the latter category of outcome measure, not least because they are weighted sums of a few binary variables themselves and were designed specifically to rank the severity of a complex phenotype most efficiently.

### DNA extraction, SNP genotyping and quality control

The SNP genotype data used in the present GWAS were generated and quality-controlled separately for Kiel and Würzburg/Berlin COVIDOM participants. DNA extraction from Kiel blood samples (*n* = 1,691) was performed at the Institute of Clinical Molecular Biology (IKMB), Kiel University, Germany, on a Chemagic 360 machine (PerkinElmer, Waltham, Massachusetts, U.S.) using low volume kit CMG-1491 and buffy coat kit CMG-714 (Chemagen, Baesweiler, Germany) according to the manufacturer protocols. Due to low buffy coat volumes, DNA extraction from the Würzburg/Berlin blood samples (*n* = 962) was performed at Helmholtz Munich (HMGU), Germany, in a manual process using standard methodology.

Genotyping was carried out at IKMB and HMGU with Illumina (Illumina Inc., San Diego, U.S.) Global Screening Array-24 Multi Disease (GSA) Version 3.0, following the Illumina Infinium HTS Assay Auto 3-day Workflow (Document #15045738v0). Genotype quality was controlled using thresholds of 0.1 for the missingness per SNP per individual and 10^− 5^ for the Hardy-Weinberg equilibrium (HWE) test p value. The latter reflects common practice to limit the effects of HWE filtering to the detection of genotyping errors while avoiding the exclusion of rare, geographically localized variants. Linkage disequilibrium-based SNP pruning was performed with a window size of 50 SNPs, a shift of five SNPs per step, and an R^2^ threshold of 0.2. The threshold for the identity-by-descent probability was 0.125.

### Genotype imputation

Missing SNPs were imputed with IMPUTE2 (version 2.3.2) using the 1000 Genomes phase 3 reference dataset divided into lots of 5 Mb each. The threshold for the Info Score was 0.7. Genotype imputation was carried out separately for the Kiel and Würzburg/Berlin data.

### Genome-wide association analysis (GWAS)

A logistic regression model as implemented in PLINK v2.00a3 SSE4.2 was used for analyzing the SNP profiles (6,383,167 variants) and various dichotomized phenotypes of 2,247 COVIDOM participants. Age, sex, virus variant (surrogate: infection before or after 27 December 2021), study site, time between infection (surrogate: PCR test) and study site visit, acute COVID-19 severity, individual resilience and the 10 first principal components of the SNP genotype profile were considered as covariates, as appropriate, and an additive SNP genotype model was employed throughout.

Inspection of the first two principal components (PCs) revealed a strong genetic similarity between COVIDOM participants and the EUR population from the 1000 Genomes project, thereby confirming the Northern and Western European ancestry of the vast majority of the former (Supplementary Fig. 1). A total of 38 participants (21 Kiel, 17 Würzburg/Berlin) were identified as outliers because of a PC1 or PC2 value that was more than five interquartile ranges below or above the 1st or 3rd quartile of the CEU population, respectively. We also assessed the potential level of genetic stratification of COVIDOM participants with regard to each of the dichotomized PCS scores, using the so-called ‘genomic inflation factor’ λ_G_^15^ as calculated with R functions *median* and *qchisq* (http://genometoolbox.blogspot.com/2014/08/how-to-calculate-genomic-inflation.html, accessed 20 March 2025). A value of λ_G_ close to unity indicates sufficient control for genetic confounding when comparing two samples of participants in a GWAS.

No formal statistical testing of a genome-wide null hypothesis (i.e. no genetic effect at all) was involved in the interpretation of the analytical results. Instead, the association signals were ranked according to their respective p values, and the threshold for ‘suggestive’ statistical significance and, hence, further consideration of an association was set to 10^− 5^ in order to balance the chances of false positive and negative findings. This notwithstanding, the statistical power of the available data was assessed with the UCSF Sample Size Calculator assuming a dominant effect of the minor SNP allele and a significance level of 10^− 5^. For a minor allele frequency (MAF) of 0.2, the data provided 90% power to detect an allelic odds ratio (OR) of 1.75 or larger, and 80% power to detect an OR of 1.65 or larger. If the MAF was 0.3, the corresponding OR limits were 1.65 (90%) and 1.55 (80%), respectively.

In addition to the SNP-wise analyses, we assessed the polygenic background of PCS by way of polygenic scores constructed for the three dichotomized PCS scores from the GWAS summary statistics. For this, we used the LDpred2 method implemented in R package *bigsnpr*^16^ with the default HapMap3+-based LD correlation matrix as provided by the package. The performance of the polygenic scores was evaluated by the Area Under Curve (AUC) obtained when treating each polygenic score as a predictor of the corresponding (dichotomized) PCS score in the underlying GWAS dataset. If one of the PCS scores had a notable polygenic background, an overperformance of the corresponding polygenic score would have been expected in this regard.

### Combinatorial analysis

For the 118 SNP genotype combinations (“disease signatures”) found by Taylor et al.^9^ to be associated with more severe or fatigue-dominant phenotypes of long COVID syndrome, carriers in COVIDOM were compared to non-carriers for their PCS severity as quantified by one of the three PCS scores, individual resilience or acute disease severity, using a Mann-Whitney test. Since each disease signature was considered five times, we applied a per-signature Bonferroni correction for multiple testing by setting the p value threshold for statistical significance to 0.05/5 = 0.01. This threshold reflects our primary intention to determine the strength of association between the PCS phenotypes considered and each individual disease signature, rather than formally testing the stringent global null hypothesis that none of the 118 disease signatures was associated with PCS.

## Results

### Data quality control

The 1,691 Kiel DNA samples were genotyped for 730,059 SNPs. Subsequent quality control led to the exclusion of 169 individuals because of unclear sex (*n* = 30), excess chance of relatedness (*n* = 85), reduced heterozygosity (*n* = 32) or likely non-European origin (*n* = 22), leaving 1,522 individuals for SNP genotype imputation. Several SNPs had to be excluded from further analyses because of excess LD to other variants (*n* = 450,139), missingness (*n* = 7,915) or lack of Hardy-Weinberg equilibrium (*n* = 3,022). After imputation and filtering with an Info Score threshold of 0.7, genotype data of Kiel COVIDOM participants were available for 5,954,344 SNPs.

For the Würzburg/Berlin sub-cohort, SNP genotypes of 836 of 962 individuals were subjected to imputation after quality control. Of the 654,027 initially included SNPs, some 265,307 remained after excluding variants for excess LD (*n* = 378,116), missingness (*n* = 10,596) or lack of Hardy-Weinberg equilibrium (*n* = 310). After imputation and filtering, genotypes of 5,792,334 SNPs from Würzburg/Berlin participants were available for further analysis.

### Characteristics of COVIDOM GWAS cohort

A total of 2,247 COVIDOM participants had sufficient genotype and phenotype data available for inclusion into at least one of the subsequent GWAS (Table 1; for the individual frequencies of the PCS score-defining symptom complexes, see Supplementary Table 1). This cohort was characterized by a mean age of 45.8 years (SD: 15.4), a proportion of females of 56.0%, and a mean BMI of 26.8 kg/m² (SD: 5.4). Some 1,112 participants (51.15%) were self-reported current or former smokers, and 138 (6.2%) required hospitalization due to severe acute COVID-19.

**Table 1:** Characteristics of COVIDOM GWAS cohort (*n* = 2,247).

Parameter	Value
Age [years]	45.8 (15.4)
Female sex	1,256 (56.0%)
BMI [kg/m^2^]	26.8 (5.4)
Hospitalization for acute COVID-19	138 (6.2%)
Smoker [current or former]	1,112 (51.15%)

Values are mean and standard deviation or number and percentage, as appropriate.

### GWAS

#### Original PCS score

A PCS score-based median split (at 15.5 points) of the combined Kiel and Würzburg/Berlin dataset yielded 1,048 cases and 1,067 controls, with no evidence of systematic genetic confounding (λ_G_ = 1.016). With this sample size, only unrealistically strong genotype-phenotype associations would have been detectable at the so-called ‘genome-wide significance level’ of 5 × 10^− 8^, which in turn would have led to an unacceptably high false negative rate. Based upon preceding power calculations, we therefore set the p value threshold for further consideration of an association as ‘suggestively’ significant to 10^− 5^.

**Fig. 1:**
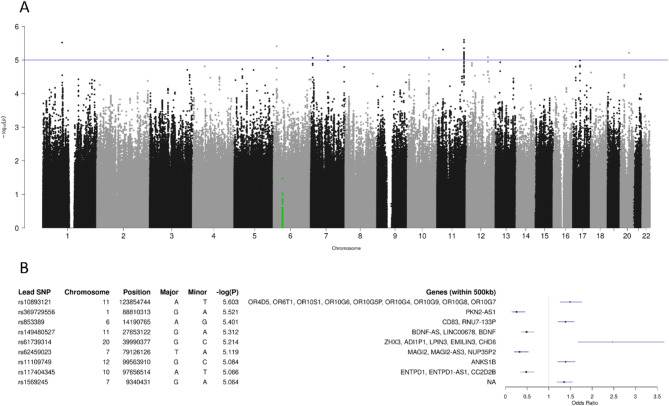
GWAS of dichotomized original PCS score (median split). (A) Manhattan plot of -log_10_(p) values. The region around the *FOXP4* gene on chromosome 6 is highlighted in green. The blue line corresponds to a suggestive significance level of 10^− 5^. (B) Lead SNPs for genotype-phenotype associations with *p* < 10^− 5^. Gene symbols are listed for genes located within 500 kb on either side of a lead SNP. Horizontal bars demarcate 95% confidence intervals for the allelic odds ratios of the minor alleles.

Under this condition, nine loci showed a suggestively significant genotypic association with the dichotomized original PCS score (Fig. 1A). All genes located within 500 kb on either side of the respective lead SNPs are shown in Fig. 1B. The most significant association, with SNP rs10893121 (*p* = 2.5 × 10^− 6^) at position chr11:123,854,744, is located in a region harbouring genes from olfactory receptor families 4, 6 and 10. The minor allele of rs10893121 increases the risk of developing more severe PCS by approximately 50% (OR = 1.49). The strongest association (OR = 2.47) was observed for SNP rs61739314 located at position chr20:39,990,377 in a region that contains the *ZHX3* (zinc fingers and homeoboxes 3), *LPIN3* (lipin 3), *EMILIN3* (elastin microfibril interfacer 3) and *CHD6* (chromodomain helicase DNA-binding protein 6) genes. Notably, the region around the *FOXP4* gene (highlighted in green in Fig. 1A), the site of the sole genome-wide significant association with PCS reported so far, by Lammi et al.^8^, was not found to be associated (at the ‘suggestive’ significance level of *p* < 10^− 5^) with the dichotomized original PCS score in our data. Moreover, none of the associations highlighted in the present GWAS achieved nominal significance in the study by Lammi et al.^8^.

To assess the polygenic component of the original PCS score, a polygenic score was constructed from the GWAS summary statistics obtained for individual SNPs. However, treatment of the polygenic score as a predictor of the dichotomized original PCS score in the underlying GWAS dataset yielded an AUC value of only 0.523, suggesting that the polygenic contribution to the PCS score was rather small.

#### Acute COVID-19 severity-specific PCS score (PCS-S)

**Fig. 2:**
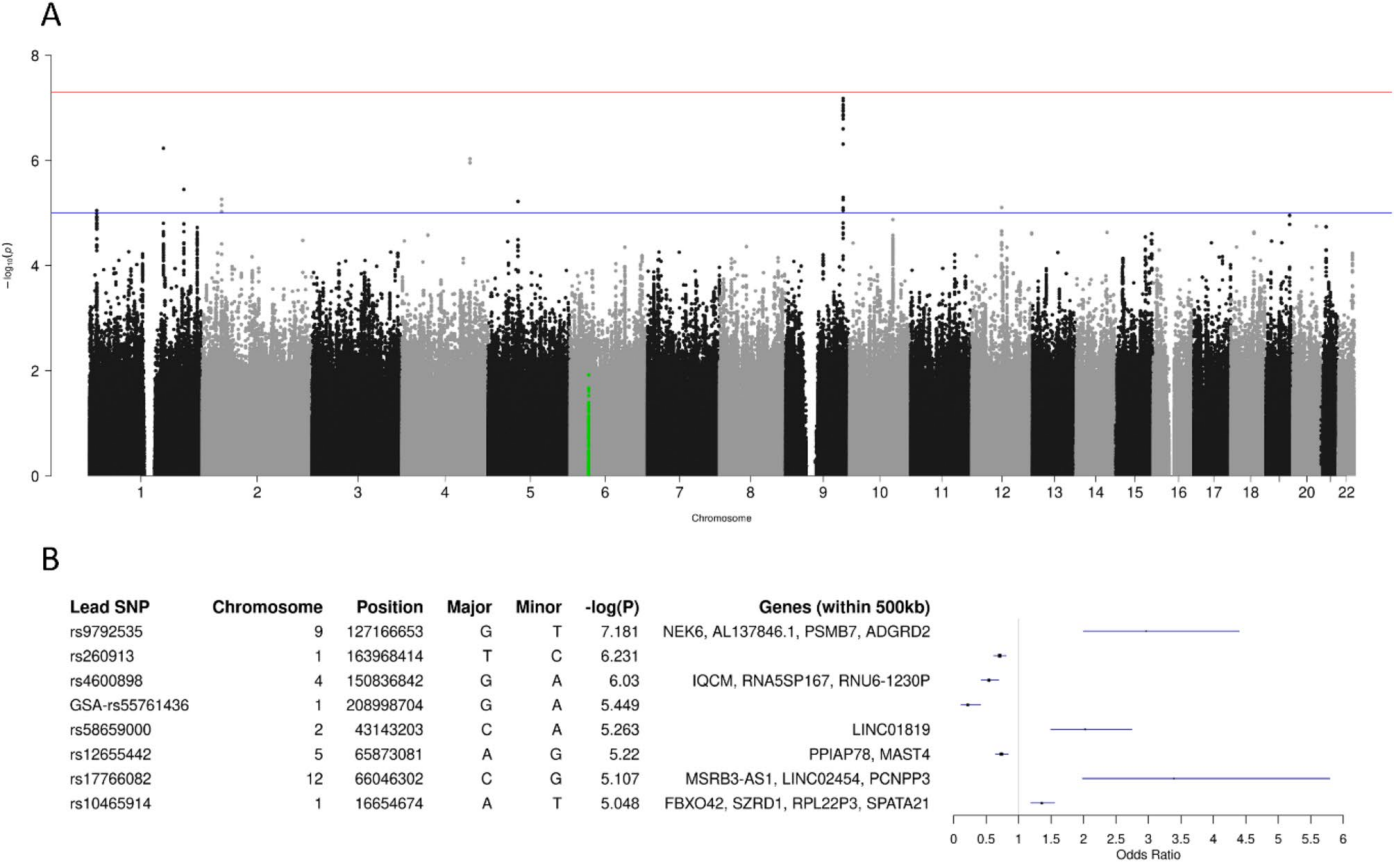
GWAS of dichotomized acute severity-specific PCS-S score (median split). (A) Manhattan plot of -log_10_(p) values. Note: With *p* = 6.6 × 10^− 8^, the PCS-S association of rs9792535 narrowly missed the threshold for genome-wide significance of 5 × 10^− 8^ (red line). (B) Lead SNPs for genotype-phenotype associations with *p* < 10^− 5^. For details, see legend to Fig. 1.

The median of the PCS-S score equalled 2.5, and the corresponding median split resulted in 1,124 controls and 1,092 cases (λ_G_ = 1.012). The most significant genotypic association with the dichotomized score was observed for SNP rs9792535 (*p* = 6.6 × 10^− 8^) located at position chr9:127,166,653 (Fig. 2A). Carriership of the minor allele of this SNP increased the risk for the more severe acute disease-specific sub-type of PCS by a factor of 3.5. Genes located in this region include *NEK6*, *PSMB7* and *ADGRD2* (Fig. 2B). Other genes around SNPs that achieved *p* < 10^− 5^ were *MAST4* on chromosome 5, *FBXO42* and *SZRD1* on chromosome 1, and pseudogene RNU6-1230P on chromosome 4 (Fig. 2B). The AUC of the PCS-S score-specific polygenic score equalled 0.503.

#### Individual resilience-specific PCS score (PCS-R)

**Fig. 3:**
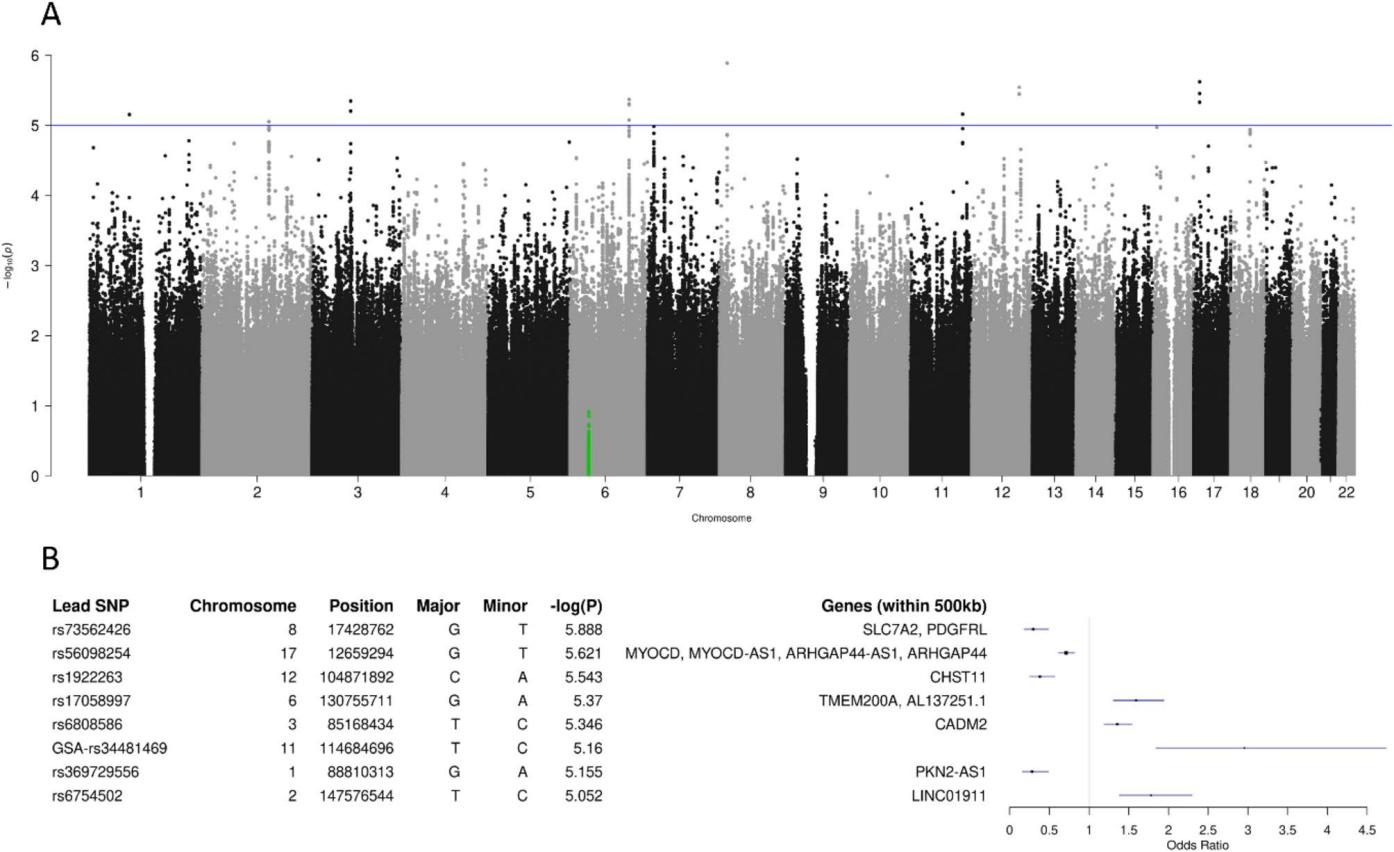
GWAS of dichotomized individual resilience-specific PCS-R score (median split). (A) Manhattan plot of -log_10_(p) values. (B) Lead SNPs for genotype-phenotype associations with *p* < 10^− 5^. For details, see legend to Fig. 1.

The median of the individual resilience-specific PCS-R score (comprising fatigue, neurological symptoms and sleep disturbance) equalled 91, and the according median split resulted in 1,095 controls and 1,076 cases (λ_G_ = 1.012). Associations between the dichotomized phenotype and the lead SNPs at eight loci achieved *p* < 10^− 5^ (Fig. 3A). Genes in the surrounding 500 kb regions (Fig. 3B) include *SLC7A2* and *PDGFRL* (chromosome 8), *MYOCD* and *ARHGAP44* (chromosome 17), *CHST11* (chromosome 12), *TMEM200A* (chromosome 6) and *CADM2* (chromosome 3). The AUC of the PCS-R score-specific polygenic score equalled 0.522.

#### Individual resilience and acute COVID-19 severity

In addition to the PCS-R and PCS-S scores, we also treated their main predictors, individual resilience and acute COVID-19 severity, as outcomes in GWAS to rule out indirect SNP genotype associations with either of the two scores. In both cases, however, no overlap was observed between regions of suggestively significant SNP genotype associations (*p* < 10^− 5^) with either a score or its specific predictor.

A suggestively significant association (*p* < 10^− 5^) with dichotomized acute COVID-19 severity (< 4 or ≥ 4 severe or life-threatening symptoms) was identified at 18 loci (Supplementary Fig. 2), with lead SNP rs78692815 at position chr11:108,617,211 narrowly missing genome-wide significance (*p* = 6.5 × 10^− 8^). Four loci showed a suggestively significant association (*p* < 10^− 5^) with dichotomized individual resilience (Supplementary Fig. 3). The most significant association was observed for SNP rs2780980 at position chr9:86,234,596 (*p* = 2.6 × 10^− 6^).

### Combinatorial analysis

In a combinatorial analysis, Taylor et al.^9^ previously identified various SNP genotype combinations (“disease signatures”) to be associated with severe PCS. We compared carriers and non-carriers of these signatures in terms of their PCS scores, individual resilience and acute COVID-19 severity. Of the 118 disease signatures tested, 12 showed a nominally significant association (*p* < 0.05) with at least one of the three dichotomized PCS scores (Table 2). However, only the association between disease signature 12 and the original PCS score remained significant after Bonferroni correction (*p* < 0.01). Notably, only six carriers of disease signature 12 were present in our dataset, five of which had an original PCS score above the median. For the remaining disease signatures, none of the associations with any of the three PCS scores was statistically significant (Supplementary Table 2).

When considering individual resilience and acute COVID-19 severity as outcome measures, nine signatures were found to be associated with at least one of the two phenotypes at the nominal significance level of 0.05 (Supplementary Table 3). However, only the association between signature 110 and individual resilience remained significant after Bonferroni correction (*p* < 0.01). None of the associations between the remaining signatures and the two phenotypes was statistically significant (Supplementary Table 4).

**Table 2:** Disease signatures (Taylor et al. 2023) showing a nominally significant association (*p* < 0.05) with at least one dichotomized PCS score.

Disease signature	SNPs	PCS score		PCS-S		PCS-*R*	
		Carrier number	*P* value	Carrier number	*P* value	Carrier number	*P* value
1	rs1933613, rs7789699, rs1427213, rs9909665	54	0.023	54	0.043	54	0.013
2	rs2795078, rs7789699, rs1427213, rs11076196	46	0.058	46	0.191	48	0.018
3	GSA.rs116450859, rs1177952, rs649305, rs2027670, rs8091293	13	0.047	13	0.326	13	0.019
4	rs17771104, rs6555852, GSA.rs10898088, rs7300070, rs4346455	42	0.050	43	0.050	43	0.024
5	rs17771104, rs6555852, GSA.rs10898088, rs7300070, rs974266	42	0.050	43	0.050	43	0.024
12	rs2379156, rs4660652, GSA.rs1532164, rs79790545, rs11568817	**6**	**0.004**	7	0.043	6	0.077
14	rs1933613, rs7789699, rs1427213, rs11076196	40	0.035	40	0.025	40	0.084
38	rs12623781, rs7789699, rs1427213, rs11076196	27	0.159	30	0.033	29	0.294
42	GSA.rs116450859, rs7789699, rs1427213, rs73016891, rs11076196	10	0.114	10	0.029	10	0.326
45	rs4657153, rs1498606, rs4715000, rs1925546, rs6082699	7	0.185	8	0.035	7	0.331
50	GSA.rs1260773, rs9497606, rs1177952, rs115758894, rs8091293	22	0.199	24	0.044	23	0.375
65	GSA.rs2073925, GSA.rs4910484, rs1191543, rs12882037, rs8038697	12	0.386	13	0.047	12	0.580

The (sole) association with a p value below the Bonferroni-corrected threshold for statistical significance (*p* < 0.01; see Methods) is highlighted.

## Discussion

Our study revealed that genetic variation at several loci throughout the human genome is potentially related to the severity of the long-term sequelae of COVID-19, which was quantified here by one of three PCS scores previously developed by our group for the work-up of the SARS-CoV-2 pandemic. As can be inferred from the Manhattan plots illustrating the GWAS results (Figs. 1, 2 and 3), however, none of the observed associations reached the stringent level of so-called ‘genome-wide significance’ (5 × 10^− 8^). Similarly, the corresponding QQ plots do not suggest strong genetic effects upon the three PCS scores studied (Supplementary Fig. 4). Although adjusting the analyses for additional covariates would reduce the p values of individual genotype-phenotype associations further, particularly when considering BMI (Supplementary Table 5), the main results would however remain unchanged in that the allelic odds ratios estimated for the minor alleles of all lead SNPs virtually stayed the same (Supplementary Table 6).

In our view, this outcome does not detract from the scientific value of the GWAS. On the contrary: Not only would non-reporting of a lack of genome-wide significant results contribute to publication bias, the information that PCS, when regarded as a composite phenotype, does not appear to have a strong genetic basis is very important to guide future research in this field.

While we could not reproduce, even at a less stringent ‘suggestive’ significance level of 10^− 5^, the single genome-wide genotype-phenotype association reported so far for PCS, namely with the *FOXP4* gene on chromosome 6^8^, we nevertheless identified SNPs in other genomic regions that are at least suggestive of a functional involvement into PCS etiology and that point towards biologically meaningful links to the phenotype in question. This particularly includes olfactory impairment in the case of rs10893121, the SNP most significantly associated (*p* = 2.5 × 10^− 6^) with the original PCS score.

Previous findings that acute COVID-19 severity and individual resilience are the sole main predictors of PCS severity previously encouraged us to develop two additional PCS scores, PCS-S and PCS-R, to address different sub-domains of PCS that are related to either predictor. While the PCS-S score comprises a number of rather different symptom complexes (impairment of smell and taste, fatigue, joint or muscle pain, general signs of infection, and exercise intolerance), the PCS-R score only included two complexes, in addition to fatigue, namely neurological ailments and sleep disturbances. Interestingly, it turned out that the potential genotypic associations with the two predictor-specific scores did not overlap with one another, providing evidence that they may indeed reflect different clinical entities. Since their constituent symptom complexes were also non-overlapping, it was not surprising that the two sub-scores were associated with different SNPs, and even although the observed associations were comparatively weak, they may be worth follow-up research to shed more light on the etiology of PCS.

PCS undoubtedly is a heterogeneous clinical condition, and the major hypotheses about PCS pathophysiology currently include (I) immune dysregulation, (II) microbiota dysbiosis, (III) autoimmunity and immune imprinting, (IV) blood clotting and endothelial abnormalities and (V) dysfunctional neurological signalling^3^. In the following, we will assess whether the potential genetic associations identified for the three PCS scores in the present GWAS would be consistent with the above hypotheses, considering possible functional links to genes located within or near the associated regions.

### PCS score

#### rs10893121: olfactory receptors OR4D5, OR6T1, OR10S1, OR10G4, OR10G7, OR10G8 and OR10G9

The perception of smell is triggered by odorant molecules in the nasal tissue that initiate a neural response. Olfactory receptors are members of a large family of G protein-coupled receptors that are responsible for the G protein-mediated transduction of olfactory sensory signals. OR4D5, OR6T1, OR10S1, OR10G4, OR10G7, OR10G8 and OR10G9 all belong to this protein family^17,18^. While their role in olfactory function is clear-defined, no particular disease has so far been associated with SNPs in the corresponding gene regions. Important in the present context, impairment of smell and taste contributes only 3.5 points to the PCS score (range: 0 to 59) and hence adds only little to the overall severity of PCS. Moreover, while impaired smell is a pathognomonic feature of acute COVID-19 that may persist for a long time, its impact on health-related quality of life is not very strong. The fact that SNP rs10893121 showed the most significant association of all with the original PCS score indicates that variation in olfactory receptor function may nevertheless be a genetic cause of longstanding olfactory impairment after (even mild) SARS-CoV-2 infection.

#### rs61739314: zinc fingers and homeoboxes 3 (ZHX3), lipin 3 (LPIN3), Elastin microfibril interfacer 3 (EMILIN3), chromodomain helicase DNA-binding protein 6 (CHD6)

The products of the *ZHX* gene family, including *ZHX3*, comprise two C2H2-typic zinc fingers and other proteins that may function as transcriptional repressors^19,20^. While there is evidence that these gene products represent (unfavourable) prognostic markers of renal, urothelial, endometrial and thyroid cancer, no associations have been reported so far to cardiovascular or inflammatory diseases^21–23^. Furthermore, no *ZHX* gene-encoded mRNA is detectable in immune cells, and the corresponding proteins are not found in blood. Therefore, a causal role of these genes in PCS pathophysiology is not very likely.

The protein encoded by the *CHD6* gene can function as a transcriptional repressor and is involved in the cellular repression of influenza virus replication^24^. This role in host-virus interaction, and the capability of the protein to activate gene transcription in response to oxidative stress through an interaction with NFE2L2, may be a plausible explanation for the association between local SNP rs61739314 and PCS^25^.

The *LPIN3* gene is ubiquitously expressed in duodenum, skin and more than 20 other tissues. One of the functions of lipin complexes is to contribute to gene regulation by acting as transcriptional co-activators in the nucleus^26,27^. In addition, lipin complexes process precursors of triglycerides and phospholipids in the cytoplasm^28^. However, protein functions that might be relevant in PCS pathophysiology are not immediately apparent.

Finally, the *EMILIN3* gene product is part of the collagen-containing extra-cellular matrix. While the gene is highly expressed in connective tissue in both female and male reproductive organs, a connection to pathophysiological processes of PCS is also not obvious^29^.

### PCS-S score

#### rs9792535 – NIMA related kinase 6 (NEK6), proteasome 20 S subunit beta 7 (PSMB7) and adhesion G protein-coupled receptor D2 (ADGRD2)

The ‘never in mitosis A’ (*NIMA*) gene of *Aspergillus nidulans* encodes a serine/threonine kinase that controls initiation of mitosis. Human NIMA-related kinases (NEKs), like NEK6, are homologues of fungal NIMA and perform similar functions. Inhibition of the proteins can lead to apoptosis^30,31^. The *NEK6* gene is most abundantly expressed in gallbladder but is neither specific to immune cells nor expressed in blood. While there are some connections between the gene and tumorigenesis, e.g. by suppressing p53-induced cancer cell senescence^32^, no link to PCS pathophysiology is obvious.

Proteasome 20 S subunit beta 7 (PSMB7) is a multi-catalytic proteinase complex that is distributed throughout eukaryotic cells and cleaves peptides in a non-lysosomal pathway. Gamma interferon may downregulate this proteosomal catalytic subunit. The PSMB7 complex plays many essential roles in the cell by associating with different regulatory particles. Removing misfolded or damaged proteins that could impair cellular function is one of the major capacities of the gene product^33,34^. Adhesion G protein-coupled receptor D2 (ADGRD2) is predicted to be involved in adenylate cyclase-activating G protein-coupled receptor signaling pathway and is an integral component of the membrane^35,36^. Therefore, an involvement of PSMB7 or ADRG2 also in processes hypothesized to cause PCS appears plausible.

#### rs58659000, rs10465914 ﻿– MAST4, FBXO42 and SZRD1

The *MAST4* gene encodes a protein belonging to the microtubule-associated serine/threonine protein kinases, which are mainly expressed in cytoplasm, predominantly in esophagus and urinary bladder^37^. FBXO42 is a member of the F-box protein family and is characterized by a 40 amino acid F-box motif. Full-length cloning of FBXO42 in a mammary library identified the so-called ‘Just one F-box and Kelch domain-containing protein’ (JFK), a critical negative regulator of p53^38^. Both FBXO42 and SZRD1 are negative markers of liver cancer. Finally, SZRD1 suppresses cell proliferation by inducing cell cycle arrest and apoptosis^39^. All three genes are thus not obviously connected to pathophysiological processes relevant for PCS onset or severity.

### PCS-R score

#### SLC7A2, PDGFRL, MYOCD, ARHGAP44, CHST11, TMEM200A and CADM2

The protein encoded by the *SLC7A2* (solute carrier family 7 member 2) gene is a cationic amino acid transporter belonging to the APC (amino acid-polyamine-organocation) family of transporters^40^. Located in the cell membrane, it is responsible for the cellular uptake of arginine, lysine and ornithine. Three transcript variants encoding different isoforms have been detected for this gene^40^. SLC7A2 may play a role in macrophage activation through its role, as a member of the cationic amino acid transporter protein family, in L-arginine transport.

The *PDGFRL* gene encodes a protein with significant amino acid sequence similarity to the ligand binding domain of platelet-derived growth factor receptor beta^41^. Mutations in this gene, and the deletion of a chromosomal segment containing this gene, are both associated with sporadic hepatocellular carcinomas, colorectal cancers and non-small cell lung cancers, suggesting that the gene product may function as a tumor suppressor^42,43^.

The protein encoded by the *MYOCD* gene is found in smooth muscle cells and cardiac muscle cells. Through forming a complex with serum response factor, it functions as a transcriptional activator of CArG box-dependent cardiac promotors^44^. The two proteins play a crucial role in cardiogenesis, urinary bladder development and the differentiation of smooth muscle cells (myogenesis)^44^. Potential clinical phenotypes associated with variation in the *MYOCD* gene include congenital megabladder, but no association to inflammatory, auto-immune or other processes relevant to PCS are known.

ARHGAP44 enables phospholipid binding activity and is involved in actin cytoskeleton dynamics for filopodia protrusion and cell migration^45^. It is predicted to play a role in several processes, including modification of dendritic spine, negative regulation of Rac protein signal transduction and regulation of plasma membrane-bounded cell projection organization. The protein is not detected in immune cells or blood. Previous studies suggested that ARHGAP44 has an indirect influence on viral-induced tetherin signaling^46^. There is also growing evidence that ORF7a and Spike act as tetherin antagonists in SARS-CoV-2 infections^47,48^, and another study suggested that SARS-CoV-2 inhibits ORF3a tetherin by trapping it in late endocytic organelles^49^.

The protein encoded by the *CHST11* gene belongs to the sulfotransferase 2 family. A chromosomal translocation, t(12;14)(q23;q32), involving this gene and IgH has been reported in a patient with B cell chronic lymphocytic leukemia^34^. Furthermore the protein is a negative marker of renal and urothelial cancer^50^. TMEM200A is predicted to be an integral component of the membrane, but its detailed function is still unknown.

The protein encoded by the *CADM2* gene belongs to the synaptic cell adhesion molecule 1 (SynCAM) family, which is part of the immunoglobulin superfamily^51^. It has a cytosolic binding site for members of the protein 4.1 family, known to interact with cytoskeletal proteins. Variants in the *CADM2* gene are associated with post-bronchodilator FEV1 and FEV1/FVC ratio, alcohol consumption, general risk-taking tendency and adventurousness, and BMI^52–54^.

### Strengths and limitations

The major strength of our GWAS is that it was based upon a prospective population-based cohort study of PCS with deep phenotyping of participants. Although the PCS scores used in our study drew upon patient self-reports, clinical characteristics of study participants, including laboratory measurements, vital signs, lung function, neurological testing, and echocardiography are available for possible follow-up analyses.

Compared to GWAS of other complex human phenotypes, the sample size of the present study (maximum *n* = 2,216) was modest, which may have resulted in limited power to detect weak to moderate genetic effects. However, by lowering the significance level accordingly, we aimed to reduce the risk of false negative results to a level that, we believe, ensured that clinically and biologically truly significant associations were unlikely to have been missed. Indeed, inspection of the PCS score-specific QQ plots obtained in the three GWAS reveals that an enrichment of such associations is conceivable at least for the acute severity-sensitive PCS-S score (Supplementary Fig. 4).

## Conclusions

We found various SNPs to be potentially associated with PCS severity, first and foremost variants in the olfactory receptor gene region. Impairment of smell and taste is a pathognomonic feature of both, acute COVID-19 and PCS, and our results suggest that this connection may have a genetic basis. Three other genotype-phenotype associations pointed towards an association between PCS and cellular virus repression (*CHD6*), activation of macrophages (*SLC7A2*) and the release of virus particles from infected cells (*ARHGAP44*). All other gene regions highlighted by our GWAS, however, did not relate to pathophysiological processes currently discussed for PCS. Therefore, and because the genotype-phenotype associations observed in our GWAS were generally not very strong, the complexity of the genetic background of PCS and its sub-domains appears to be rather high, and thus comparable to that of many other multifactorial traits in humans.

## Electronic supplementary material

Below is the link to the electronic supplementary material.

Supplementary Material 1

## Data Availability

All data of this study are available upon request from the NAPKON Data Use and Access Committee. For information on the NAPKON data governance and for submission of research proposals, access https://proskive.napkon.de (accessed 20 March 2025). Summary statistics of all GWAS conducted in this study are publicly available at locuszoom.org under study label ‘NAPKON POP’ (reference numbers 769746, 372109, 667841, 447780, 114850).
