# Supplementary material for: Genome-wide association study of post COVID-19 syndrome in a population-based cohort in Germany
Source: Sci Rep. 2025 May 6;15:15791. doi: 10.1038/s41598-025-00945-z (PMC12056214; doi:10.1038/s41598-025-00945-z)
Supplement: Supplementary file 1 — Supplementary Material 1 [file 41598_2025_945_MOESM1_ESM.docx]

Anne-Kathrin Ruß et al.: Genome-wide Association Study of Post COVID-19 Syndrome in a Population-based Cohort in Germany

Supplementary Material


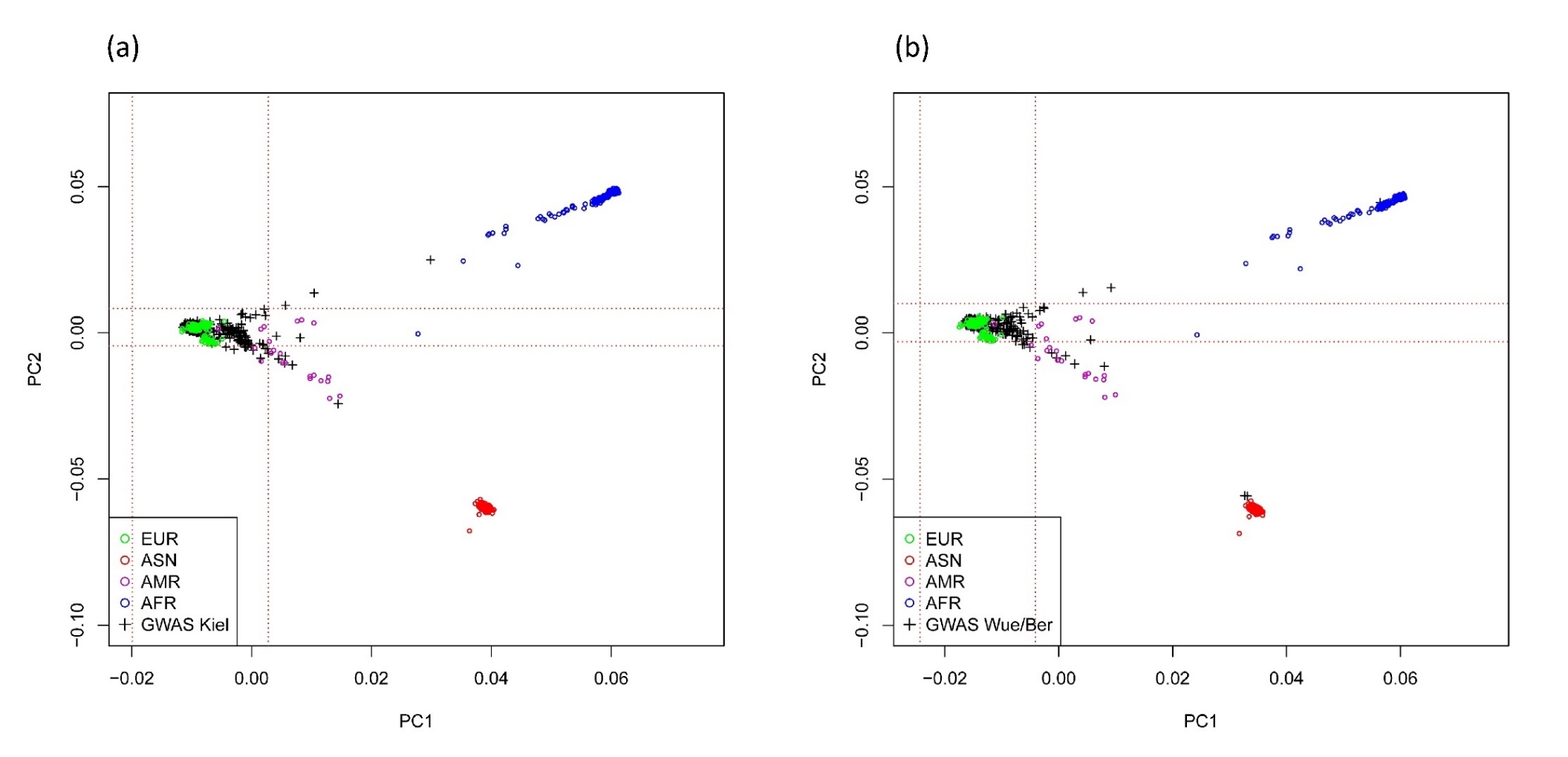


**Supplementary Figure 1. SNP genotype-based PCA of GWAS and 1000 Genomes samples.** A joint principal component analysis (PCA) was conducted of SNP genotype data from the 1000 Genomes project, including individuals of American (AMR), Asian (ASN), African (AFR) and European (EUR) ancestry, and from the present GWAS. PCA was conducted separately for Kiel (a) and Würzburg/Berlin (b) samples. Dashed red lines demarcate the thresholds used of for outlier definition (1^st^ and 3^rd^ quartile of each PC in EUR samples, plus or minus five times the respective interquartile range).

**
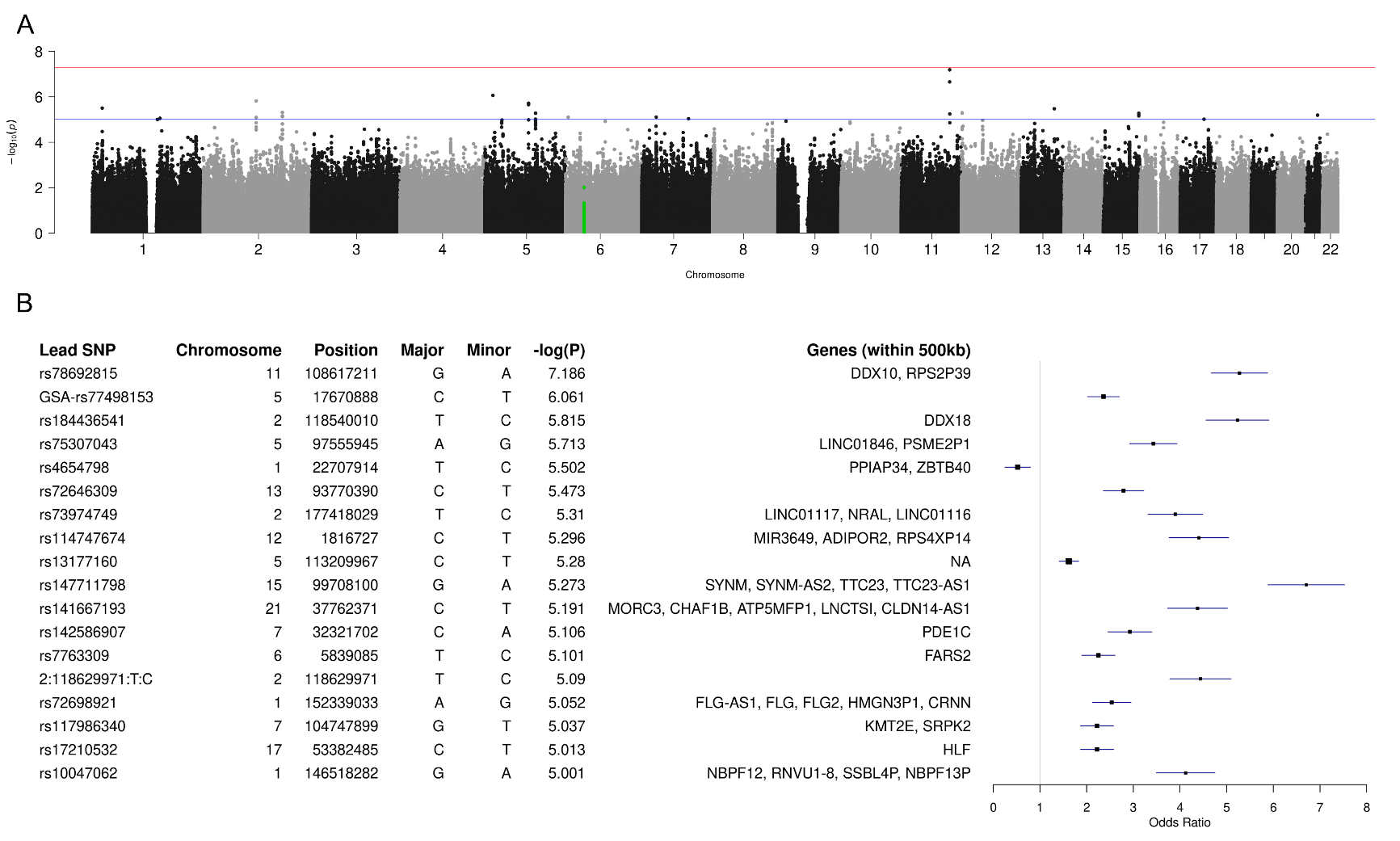
**

**Supplementary Figure 2. GWAS of dichotomized acute COVID-19 severity.** The data were split according to the number of acute COVID-19 symptoms (<4 or ≥4) that the COVIDOM study participants themselves reported as either severe or life-threatening. For details, see legend to Figure 1 of the main text.

^
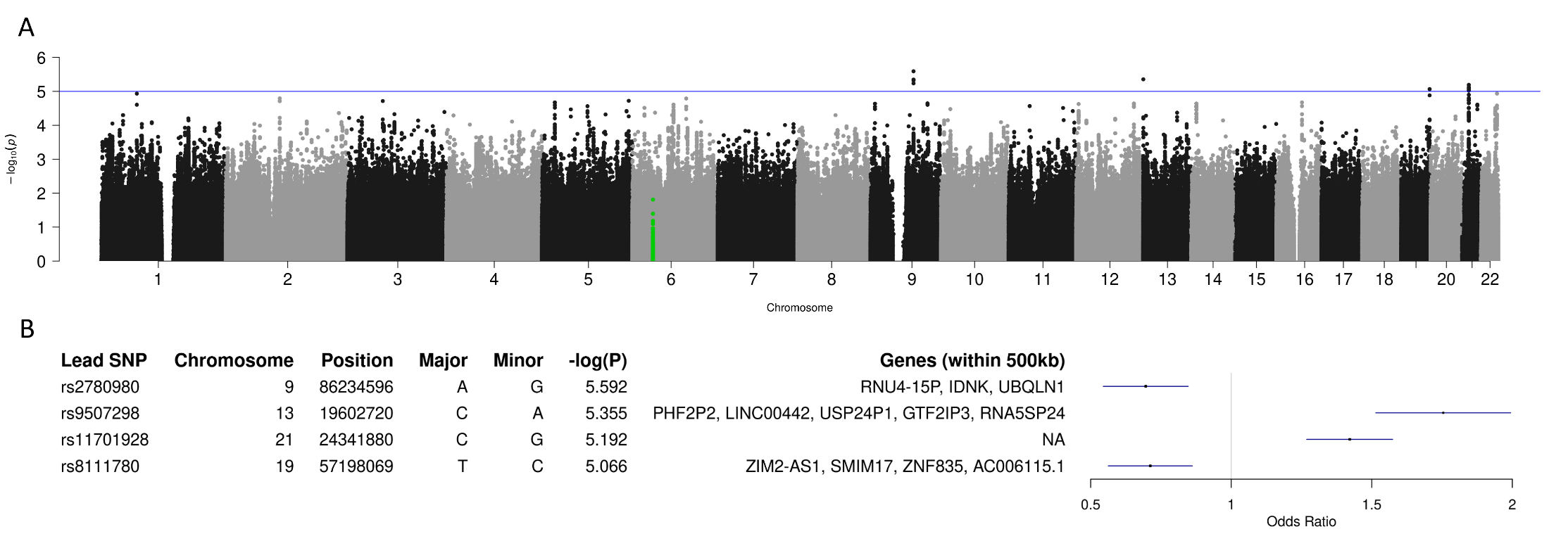
^

**Supplementary Figure 3.** **GWAS of dichotomized individual resilience.** The data were split along the median (3.67) of the BRS score. For details see, legend to Figure 1 of the main text.


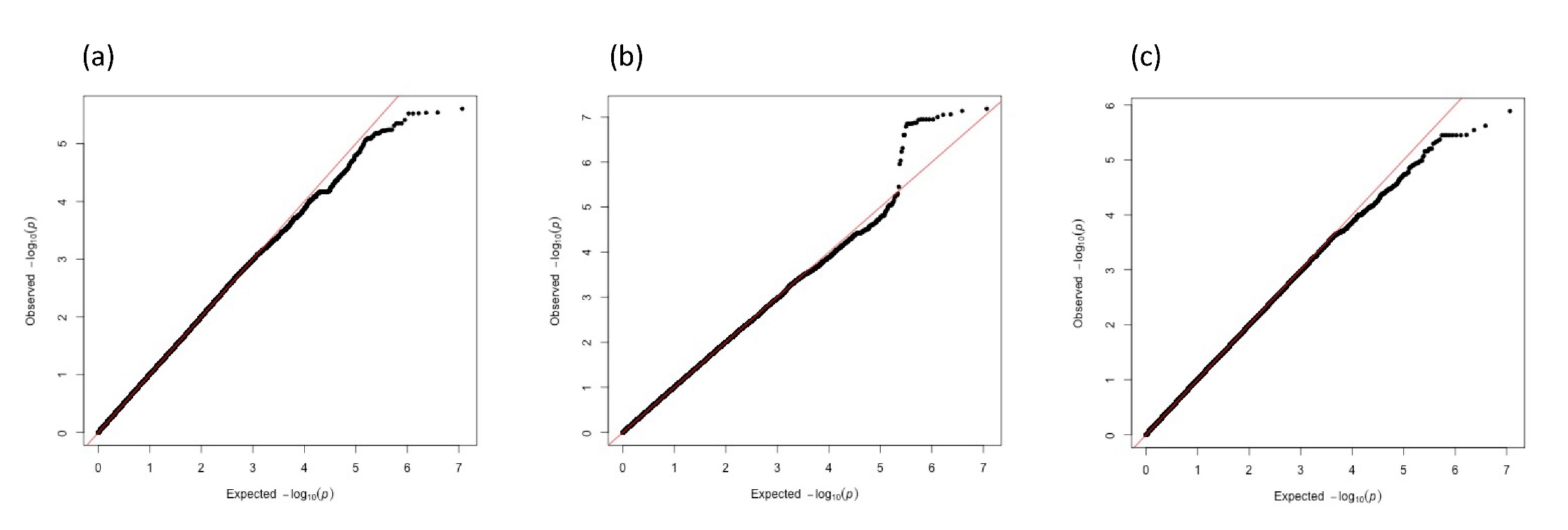
 **Supplementary Figure 4. QQ plots from GWAS of three dichotomized PCS scores.** Plots refer to p values obtained in the analysis of the original PCS score (a), the PCS-S score (b) and the PCS-R score (c), respectively.

**Supplementary Table 1. Frequencies, among participants of the COVIDOM study (n=2,247), of 12 binary symptom complexes underlying the definition of PCS scores**

| **No.** | **Symptom complex** | **Self-reported sub-symptoms** | **Absolute frequency (%)** |
| --- | --- | --- | --- |
| 1 | Chemosensory deficits | Smelling disturbance, impaired sense of taste | 416 (18.5) |
| 2 | Fatigue | Fatigue | 1263 (56.6) |
| 3 | Exercise intolerance | Shortness of breath, reduced exercise capacity | 776 (34.5) |
| 4 | Joint or muscle pain | Muscle pain, joint pain | 271 (12.1) |
| 5 | Ear-Nose-Throat (ENT) ailments | Hoarseness, sore throat, running nose | 143 (6.4) |
| 6 | Coughing, wheezing | Coughing, wheezing | 153 (6.8) |
| 7 | Chest pain | Chest pain | 146 (6.5) |
| 8 | Gastrointestinal ailments | Stomach pain, diarrhoea, vomiting, nausea | 105 (4.7) |
| 9 | Neurological ailments | Confusion, vertigo, headache, motor deficits, sensory deficits, numbness, tremor, deficits of concentration, cognition or speech | 1374 (62.7) |
| 10 | Dermatological ailments | Hair loss, rash, itchiness | 102 (4.7) |
| 11 | Infection signs | Chills, fever, general sickness/flu-like symptoms | 331 (14.9) |
| 12 | Sleep disturbance | Insomnia, unrestful sleep | 1270 (57.3) |

adapted from Bahmer et al. 2022 (reference [4] in the main text)

**Supplementary Table 2. Non-significant associations (p≥0.05) between disease signatures (Taylor et al. 2023, reference [9] in main text) and three dichotomized PCS scores**

| Disease signature | SNPs | PCS score | | PCS-S | | PCS-R | |
| --- | --- | --- | --- | --- | --- | --- | --- |
|  |  | **Carrier number** | **P value** | **Carrier number** | **P value** | **Carrier number** | **P value** |
| 6 | GSA.rs1109968, rs1925546, rs4968776, GSA.rs79745298, rs11089788 | 34 | 0.105 | 35 | 0.223 | 35 | 0.055 |
| 7 | GSA.rs58438895, rs1925546, rs4968776, GSA.rs79745298, rs11089788 | 34 | 0.105 | 35 | 0.223 | 35 | 0.055 |
| 8 | GSA.rs7566840, rs17417442, rs4755910, rs1036004, rs412830 | 39 | 0.156 | 40 | 0.157 | 40 | 0.055 |
| 9 | rs9817112, rs13134490, GSA.rs7342161, rs17106075, rs6277 | 38 | 0.066 | 39 | 0.145 | 38 | 0.055 |
| 10 | rs1883441, rs11241791, rs909975, GSA.rs28750191 | 46 | 0.055 | 48 | 0.080 | 47 | 0.059 |
| 11 | rs11756157, rs200041397, rs2237562, rs1549759, rs5880 | 3 | 0.307 | 3 | 0.767 | 3 | 0.061 |
| 13 | rs2798353, rs6431850, rs1124479, rs7300070, rs4759440 | 2 | 0.064 | 2 | 0.138 | 2 | 0.079 |
| 15 | GSA.rs1378716, rs4145309, rs9864772, GSA.rs72715247, rs2210270 | 27 | 0.139 | 27 | 0.350 | 27 | 0.090 |
| 16 | rs12616464, rs10834677, rs8015138, GSA.rs9620727 | 46 | 0.299 | 48 | 0.114 | 47 | 0.115 |
| 17 | rs6796096, rs1110350, rs12153720, rs4843458, rs1401795 | 42 | 0.284 | 42 | 0.349 | 42 | 0.121 |
| 18 | rs16861205, rs11768441, rs948194 | 70 | 0.533 | 73 | 0.930 | 70 | 0.121 |
| 19 | rs1335532, rs56231259, GSA.rs72715247, rs4534479, rs7278504 | 4 | 0.209 | 4 | 0.099 | 4 | 0.125 |
| 20 | rs6716743, GSA.rs7871649, rs6489046, rs1818710, GSA.rs460917 | 6 | 0.173 | 6 | 0.467 | 6 | 0.126 |
| 21^a^ | rs7970079, rs148551387, rs9602200, rs2572164, 16:16291099 | 19 | 0.189 | 19 | 0.522 | 19 | 0.136 |
| 22 | GSA.rs12623176, rs1875799, GSA.rs113859447, rs78498450, rs117531661 | 3 | 0.156 | 3 | 0.336 | 3 | 0.136 |
| 23 | GSA.rs116450859, GSA.rs10759982, rs1521426, rs17193509, rs233314 | 14 | 0.249 | 14 | 0.982 | 15 | 0.163 |
| 24 | rs462779, rs36097424, rs115758894 | 82 | 0.996 | 84 | 0.412 | 84 | 0.172 |
| 25 | rs3729547, rs16880691, rs17106075, rs10405246, rs11672440 | 18 | 0.227 | 18 | 0.468 | 18 | 0.179 |
| 26 | rs3729547, rs16880691, rs17106075, rs10421768, rs11672440 | 18 | 0.228 | 18 | 0.469 | 18 | 0.179 |
| 27 | rs867633, rs58902662, GSA.rs4777581, rs115758894 | 29 | 0.570 | 30 | 0.844 | 30 | 0.186 |
| 28 | rs10866290, GSA.rs2073925, rs35112391, rs2346943, rs17771175 | 5 | 0.261 | 6 | 0.632 | 5 | 0.189 |
| 29 | rs1335532, GSA.rs72715247, rs4534479, rs11197616, rs7278504 | 5 | 0.367 | 5 | 0.745 | 5 | 0.198 |
| 30 | rs67302518, GSA.rs2582469, GSA.rs1811338, rs2647676, rs1507477 | 5 | 0.311 | 6 | 0.212 | 6 | 0.203 |
| 31 | rs67302518, rs2253765, GSA.rs1811338, rs2647676, rs1507477 | 5 | 0.311 | 6 | 0.212 | 6 | 0.203 |
| 32 | rs401758, rs62386725, GSA.rs58438895, rs1925546, rs1874490 | 6 | 0.137 | 6 | 0.141 | 6 | 0.203 |
| 33 | rs201492944, rs4529355, rs948194, rs17106075, rs59481932 | 2 | 0.237 | 2 | 0.298 | 2 | 0.267 |
| 34 | rs17075031, rs4529355, rs948194, rs17106075, rs59481932 | 2 | 0.237 | 2 | 0.298 | 2 | 0.267 |
| 35 | rs6432496, rs701662, rs16970500, rs5880, rs34748139 | 2 | 0.369 | 2 | 0.298 | 2 | 0.267 |
| 36 | GSA.rs11131168, rs28670049, rs7775474, rs9490546, rs76493570 | 38 | 0.227 | 38 | 0.171 | 38 | 0.283 |
| 37 | GSA.rs2513345, rs28368589, GSA.rs1341032, rs61851154, rs4755910 | 54 | 0.433 | 56 | 0.242 | 56 | 0.284 |
| 39 | GSA.rs765786, rs533857, GSA.rs873601, rs752092, rs115758894 | 26 | 0.154 | 28 | 0.074 | 27 | 0.301 |
| 40 | rs2077753, rs13131918, rs737522 | 402 | 0.415 | 420 | 0.692 | 411 | 0.317 |
| 41 | rs7531555, rs10800790, rs6716743, rs1120653, GSA.rs1768885 | 9 | 0.387 | 9 | 0.373 | 9 | 0.321 |
| 43 | GSA.rs11131168, rs28670049, rs7775474, rs9490546, rs76491632 | 36 | 0.289 | 36 | 0.314 | 36 | 0.327 |
| 44 | rs2038486, GSA.rs116450859, rs12913328, rs12151280, rs13053354 | 5 | 0.341 | 5 | 0.398 | 5 | 0.329 |
| 46^a^ | rs7593130, rs9864288, rs4554825, rs9788816, 17:48719848 | 37 | 0.409 | 39 | 0.749 | 38 | 0.341 |
| 47 | rs2003780, rs11249632, rs11255643, rs2148073, rs12922740 | 14 | 0.554 | 16 | 0.278 | 16 | 0.353 |
| 48 | GSA.rs6777173, rs4739119, GSA.rs749377, rs2915409, rs674155 | 5 | 0.710 | 5 | 0.966 | 5 | 0.361 |
| 49 | rs1836767, GSA.rs116450859, rs7789699, rs1427213, rs11076196 | 8 | 0.298 | 8 | 0.515 | 8 | 0.374 |
| 51^a^ | rs4322881, rs1039938, GSA.rs72715247, rs8044861, rs7228894 | 68 | 0.647 | 69 | 0.915 | 70 | 0.387 |
| 52 | rs401758, rs4921028, rs9296672, GSA.rs1109968, rs1925546 | 9 | 0.700 | 9 | 0.283 | 9 | 0.392 |
| 53 | rs401758, rs4921028, rs9296672, GSA.rs58438895, rs1925546 | 9 | 0.700 | 9 | 0.283 | 9 | 0.392 |
| 54 | GSA.rs10121074, rs7148304, rs12151280, GSA.rs2015901 | 24 | 0.205 | 26 | 0.175 | 25 | 0.411 |
| 55 | rs11695861, rs6970262, rs2374315, GSA.rs4743997, rs60172187 | 16 | 0.463 | 19 | 0.607 | 16 | 0.419 |
| 56 | rs2272803, rs12408203, GSA.rs1461338, rs2016461 | 21 | 0.839 | 21 | 0.564 | 21 | 0.423 |
| 57 | rs56326015, GSA.rs765786, rs533857, GSA.rs873601, rs752092 | 13 | 0.765 | 14 | 0.493 | 14 | 0.432 |
| 58 | rs1110350, GSA.rs1332865, rs4843458, rs12450045, rs1401795 | 76 | 0.574 | 81 | 0.348 | 79 | 0.475 |
| 59 | rs342420, rs61401108, rs115758894, rs2337969, GSA.rs6010063 | 11 | 0.517 | 11 | 0.588 | 11 | 0.492 |
| 60 | rs2010963, GSA.rs2496495, rs115758894 | 113 | 0.634 | 115 | 0.350 | 116 | 0.497 |
| 61 | rs3729547, rs16880691, GSA.rs75038170, rs10405246, rs11672440 | 9 | 0.745 | 9 | 0.997 | 9 | 0.511 |
| 62 | rs3729547, rs16880691, GSA.rs75038170, rs10421768, rs11672440 | 9 | 0.746 | 9 | 0.997 | 9 | 0.511 |
| 63 | rs342096, rs75681130, rs9347553, rs17106075, GSA.rs17755657 | 9 | 0.577 | 10 | 0.693 | 9 | 0.524 |
| 64 | rs7789699, GSA.rs10121074, rs1427213, rs11076196 | 37 | 0.363 | 38 | 0.512 | 38 | 0.543 |
| 66 | rs6889348, GSA.rs7817160, rs12225758, rs1558508, rs17101923 | 5 | 0.280 | 5 | 0.538 | 5 | 0.600 |
| 67 | GSA.rs58438895, rs2814338, rs2357401, rs28377784, rs729533 | 2 | 0.784 | 2 | 0.390 | 2 | 0.604 |
| 68 | rs4309752, GSA.rs58438895, rs10766134, rs2239068, rs1403057 | 23 | 0.378 | 24 | 0.765 | 23 | 0.611 |
| 69 | GSA.rs9469341, rs1177952, rs17106075, rs2027670, rs8091293 | 13 | 0.785 | 15 | 0.814 | 13 | 0.628 |
| 70 | rs13034507, rs11681751, rs6897267, rs10842964, rs60172187 | 4 | 0.555 | 5 | 0.847 | 4 | 0.632 |
| 71 | GSA.rs116450859, rs7789699, rs16925478, rs1427213, rs11076196 | 5 | 0.908 | 5 | 0.538 | 7 | 0.659 |
| 72 | rs1261411, rs3813477, GSA.rs2073925, rs327034, rs1858993 | 7 | 0.988 | 7 | 0.621 | 7 | 0.659 |
| 73 | rs1261411, rs3813477, GSA.rs2073925, rs327025, rs1858993 | 7 | 0.988 | 7 | 0.621 | 7 | 0.659 |
| 74 | rs12534437, GSA.rs1811338, GSA.rs35136666, rs487908, rs5758036 | 9 | 0.589 | 9 | 0.336 | 9 | 0.665 |
| 75 | GSA.rs765786, rs533857, GSA.rs873601, rs752092, rs5880 | 17 | 0.161 | 17 | 0.079 | 17 | 0.667 |
| 76 | rs3729547, GSA.rs116450859, rs4625, GSA.rs1714821, rs73150887 | 15 | 0.996 | 15 | 0.347 | 15 | 0.687 |
| 77 | GSA.rs6799023, rs6901613, GSA.rs2709803, rs4986790 | 29 | 0.622 | 29 | 0.691 | 29 | 0.690 |
| 78 | GSA.rs78939910, rs4657153, rs1498606, rs4715000, rs1925546 | 7 | 0.623 | 8 | 0.885 | 7 | 0.702 |
| 79 | rs6716743, rs12492247, rs13096228, GSA.rs58184535, rs6010882 | 5 | 0.929 | 5 | 0.324 | 5 | 0.702 |
| 80 | rs6716743, rs12492247, rs13096228, GSA.rs67518897, rs6010882 | 5 | 0.929 | 5 | 0.324 | 5 | 0.702 |
| 81 | rs66579885, rs1409961, rs6575815, rs8095535, rs7508763 | 8 | 0.405 | 9 | 0.463 | 9 | 0.714 |
| 82 | rs2272803, rs12698097, GSA.rs1361028, rs17272288 | 7 | 0.655 | 7 | 0.715 | 7 | 0.722 |
| 83 | rs2385273, rs1902819, GSA.rs2151418, rs11253227, rs12624956 | 5 | 0.512 | 5 | 0.695 | 5 | 0.727 |
| 84 | GSA.rs1936980, rs1902819, GSA.rs2151418, rs11253227, rs12624956 | 19 | 0.590 | 20 | 0.692 | 19 | 0.739 |
| 85 | GSA.rs765786, rs533857, GSA.rs58438895, GSA.rs873601, rs752092 | 16 | 0.773 | 16 | 0.950 | 16 | 0.743 |
| 86 | rs9383658, rs13249564, rs1481687, rs646410 | 14 | 0.661 | 14 | 0.244 | 14 | 0.743 |
| 87 | GSA.rs315051, rs12497518, rs77626328, rs7823625, GSA.rs72715247 | 11 | 0.651 | 11 | 0.152 | 11 | 0.745 |
| 88 | rs9542920, rs177387, rs2572164, rs5880, rs4814851 | 19 | 0.775 | 19 | 0.734 | 19 | 0.747 |
| 89 | rs55717234, GSA.rs10796175, rs2761884, rs115758894 | 70 | 0.763 | 73 | 0.744 | 71 | 0.751 |
| 90 | rs2178740, rs11897873, rs202064888, GSA.rs1109968, rs11079309 | 21 | 0.852 | 21 | 0.815 | 21 | 0.781 |
| 91 | rs2178740, rs11897873, rs202064888, GSA.rs58438895, rs11079309 | 21 | 0.852 | 21 | 0.815 | 21 | 0.781 |
| 92 | rs4760420, rs589258, rs209462, rs56697205 | 55 | 0.671 | 58 | 0.818 | 55 | 0.785 |
| 93 | rs10800064, GSA.rs13061104, GSA.rs112932700, GSA.rs7971281, rs16970500 | 8 | 0.894 | 8 | 0.362 | 8 | 0.804 |
| 94 | rs2244632, rs7539947, rs13310795, GSA.rs72715247, rs10848660 | 6 | 0.990 | 7 | 0.414 | 6 | 0.804 |
| 95 | rs706439, rs11736072, rs583453, GSA.rs72715247, rs35187613 | 2 | 0.870 | 2 | 0.727 | 2 | 0.829 |
| 96 | GSA.rs7592630, rs326191, rs7300070, GSA.rs2715803, rs6500650 | 15 | 0.557 | 16 | 0.641 | 16 | 0.831 |
| 97 | rs2794828, rs62186762, rs909017, rs11568817, rs12600608 | 13 | 0.899 | 15 | 0.712 | 14 | 0.857 |
| 98 | rs2794828, rs62186762, rs909017, rs9361235, rs12600608 | 13 | 0.899 | 15 | 0.712 | 14 | 0.857 |
| 99 | GSA.rs1532164, rs11756157, rs200041397, rs2237562, rs1549759 | 6 | 0.942 | 6 | 0.804 | 6 | 0.858 |
| 100 | rs12989855, rs1531136, rs7754832, rs13273088, rs2915409 | 4 | 0.955 | 4 | 0.671 | 4 | 0.890 |
| 101 | rs6834709, rs6947745, rs7006666, rs4554825, rs750456 | 14 | 0.978 | 15 | 0.622 | 14 | 0.893 |
| 102 | GSA.rs116450859, rs7641634, rs116122098, rs835760, rs72728737 | 8 | 0.658 | 8 | 0.741 | 8 | 0.901 |
| 103 | rs156168, GSA.rs12001512, rs589258, rs8076171 | 65 | 0.922 | 67 | 0.801 | 67 | 0.902 |
| 104 | rs11695861, rs6970262, rs2374315, GSA.rs4743997, rs17106075 | 29 | 0.437 | 31 | 0.494 | 29 | 0.903 |
| 105 | rs2180419, GSA.rs10503490, rs11113367, GSA.rs75038170, rs55846612 | 7 | 0.663 | 7 | 0.634 | 7 | 0.923 |
| 106 | GSA.rs765620, rs7715703, rs115758894 | 158 | 0.965 | 169 | 0.507 | 164 | 0.947 |
| 107 | rs7593130, rs4364244, rs1820075, rs2031658, rs8131126 | 31 | 0.518 | 34 | 0.467 | 32 | 0.966 |
| 108 | rs4765687, rs11059915, GSA.rs75038170, rs1333416, rs9676118 | 2 | 0.760 | 2 | 0.628 | 2 | 0.991 |
| 109 | GSA.rs7681936, rs4554825, rs3741260, rs10765692, rs4613146 | 10 | 0.424 | 10 | 0.617 | 10 | 0.996 |
| 110 | rs9817112, GSA.rs12686279, GSA.rs7342161, rs17106075, rs6277 | 20 | 0.670 | 23 | 0.674 | 21 | 0.996 |
| 111 | rs12701653, rs17106075, rs7320516, rs11072524, GSA.rs72800214 | 0 | 1.000 | 0 | 1.000 | 0 | 1.000 |
| 112 | rs4592842, rs10935496, GSA.rs1532164, rs17098351, rs6142656 | 0 | 1.000 | 0 | 1.000 | 0 | 1.000 |
| 113 | rs7531501, rs7148304, rs12454570, rs12151280, GSA.rs2015901 | 0 | 1.000 | 0 | 1.000 | 0 | 1.000 |
| 114 | rs7531501, rs7148304, rs12454570, rs12151280, rs4381836 | 0 | 1.000 | 0 | 1.000 | 0 | 1.000 |
| 115 | rs201492944, rs4529355, GSA.rs76996768, rs948194, rs17106075 | 0 | 1.000 | 0 | 1.000 | 0 | 1.000 |
| 116 | rs17075031, rs4529355, GSA.rs76996768, rs948194, rs17106075 | 0 | 1.000 | 0 | 1.000 | 0 | 1.000 |
| 117 | GSA.rs2017143, rs847622, rs4896307, rs1485534, rs7950336 | 0 | 1.000 | 0 | 1.000 | 0 | 1.000 |
| 118 | GSA.rs2017143, rs847622, rs4896307, rs7950336, GSA.rs35136666 | 0 | 1.000 | 0 | 1.000 | 0 | 1.000 |

^a^at least one SNP included in the original definition of the disease signature missing

**Supplementary Table 3. Disease signatures (Taylor et al. 2023, reference [9] in main text) showing a nominally significant association (p<0.05) with individual resilience or acute COVID-19 severity.**

| Disease signature | SNPs | Individual resilience^b^ | | Acute COVID-19 severity^c^ | |
| --- | --- | --- | --- | --- | --- |
|  |  | **Carrier number** | **P value** | **Carrier number** | **P value** |
| 4 | rs17771104, rs6555852, GSA.rs10898088, rs7300070, rs4346455 | 27 | 0.373 | 43 | 0.039 |
| 5 | rs17771104, rs6555852, GSA.rs10898088, rs7300070, rs974266 | 27 | 0.373 | 43 | 0.039 |
| 9 | rs9817112, rs13134490, GSA.rs7342161, rs17106075, rs6277 | 30 | 0.011 | 39 | 0.691 |
| 23 | GSA.rs116450859, GSA.rs10759982, rs1521426, rs17193509, rs233314 | 10 | 0.244 | 15 | 0.041 |
| 28 | rs10866290, GSA.rs2073925, rs35112391, rs2346943, rs17771175 | 1 | 0.649 | 6 | 0.047 |
| 42 | GSA.rs116450859, rs7789699, rs1427213, rs73016891, rs11076196 | 6 | 0.091 | 10 | 0.034 |
| 78 | GSA.rs78939910, rs4657153, rs1498606, rs4715000, rs1925546 | 6 | 0.427 | 9 | 0.040 |
| 96 | GSA.rs7592630, rs326191, rs7300070, GSA.rs2715803, rs6500650 | 12 | 0.224 | 17 | 0.015 |
| 110 | rs9817112, GSA.rs12686279, GSA.rs7342161, rs17106075, rs6277 | **16** | **0.002** | 23 | 0.419 |

The (sole) association below the Bonferroni-corrected threshold for statistical significance (p<0.01) is highlighted. ^a^at least one SNP included in the original definition of the disease signature missing; ^b^dichotomized along the median (3.67) of the BRS score; ^c^dichotomized along the number of acute COVID-19 symptoms (<4 or ≥4) self-reported as either severe or life-threatening

**Supplementary Table 4. Non-significant associations (p≥0.05) between disease signatures (Taylor et al. 2023) and individual resilience or acute COVID-19 severity.**

| Disease signature | SNPs | Individual resilience^b^ | | Acute COVID-19 severity^c^ | |
| --- | --- | --- | --- | --- | --- |
|  |  | **Carrier number** | **P value** | **Carrier number** | **P value** |
| 1 | rs1933613, rs7789699, rs1427213, rs9909665 | 32 | 0.080 | 54 | 0.393 |
| 2 | rs2795078, rs7789699, rs1427213, rs11076196 | 24 | 0.726 | 49 | 0.401 |
| 3 | GSA.rs116450859, rs1177952, rs649305, rs2027670, rs8091293 | 10 | 0.146 | 13 | 0.356 |
| 6 | GSA.rs1109968, rs1925546, rs4968776, GSA.rs79745298, rs11089788 | 30 | 0.243 | 35 | 0.344 |
| 7 | GSA.rs58438895, rs1925546, rs4968776, GSA.rs79745298, rs11089788 | 30 | 0.243 | 35 | 0.344 |
| 8 | GSA.rs7566840, rs17417442, rs4755910, rs1036004, rs412830 | 32 | 0.776 | 40 | 0.926 |
| 10 | rs1883441, rs11241791, rs909975, GSA.rs28750191 | 35 | 0.649 | 50 | 0.329 |
| 11 | rs11756157, rs200041397, rs2237562, rs1549759, rs5880 | 2 | 0.641 | 3 | 0.926 |
| 12 | rs2379156, rs4660652, GSA.rs1532164, rs79790545, rs11568817 | 6 | 0.660 | 7 | 0.199 |
| 13 | rs2798353, rs6431850, rs1124479, rs7300070, rs4759440 | 1 | 0.703 | 2 | 0.863 |
| 14 | rs1933613, rs7789699, rs1427213, rs11076196 | 25 | 0.533 | 40 | 0.243 |
| 15 | GSA.rs1378716, rs4145309, rs9864772, GSA.rs72715247, rs2210270 | 20 | 0.968 | 27 | 0.332 |
| 16 | rs12616464, rs10834677, rs8015138, GSA.rs9620727 | 31 | 0.277 | 48 | 0.999 |
| 17 | rs6796096, rs1110350, rs12153720, rs4843458, rs1401795 | 27 | 0.902 | 42 | 0.495 |
| 18 | rs16861205, rs11768441, rs948194 | 44 | 0.650 | 73 | 0.924 |
| 19 | rs1335532, rs56231259, GSA.rs72715247, rs4534479, rs7278504 | 3 | 0.533 | 4 | 0.633 |
| 20 | rs6716743, GSA.rs7871649, rs6489046, rs1818710, GSA.rs460917 | 6 | 0.807 | 6 | 0.548 |
| 21^a^ | rs7970079, rs148551387, rs9602200, rs2572164, 16:16291099 | 13 | 0.869 | 19 | 0.779 |
| 22 | GSA.rs12623176, rs1875799, GSA.rs113859447, rs78498450, rs117531661 | 3 | 0.800 | 3 | 0.683 |
| 24 | rs462779, rs36097424, rs115758894 | 58 | 0.464 | 84 | 0.155 |
| 25 | rs3729547, rs16880691, rs17106075, rs10405246, rs11672440 | 13 | 0.085 | 18 | 0.377 |
| 26 | rs3729547, rs16880691, rs17106075, rs10421768, rs11672440 | 13 | 0.085 | 18 | 0.377 |
| 27 | rs867633, rs58902662, GSA.rs4777581, rs115758894 | 23 | 0.507 | 31 | 0.891 |
| 29 | rs1335532, GSA.rs72715247, rs4534479, rs11197616, rs7278504 | 3 | 0.529 | 5 | 0.106 |
| 30 | rs67302518, GSA.rs2582469, GSA.rs1811338, rs2647676, rs1507477 | 3 | 0.720 | 6 | 0.104 |
| 31 | rs67302518, rs2253765, GSA.rs1811338, rs2647676, rs1507477 | 3 | 0.720 | 6 | 0.104 |
| 32 | rs401758, rs62386725, GSA.rs58438895, rs1925546, rs1874490 | 2 | 0.672 | 6 | 0.644 |
| 33 | rs201492944, rs4529355, rs948194, rs17106075, rs59481932 | 2 | 0.319 | 2 | 0.061 |
| 34 | rs17075031, rs4529355, rs948194, rs17106075, rs59481932 | 2 | 0.319 | 2 | 0.061 |
| 35 | rs6432496, rs701662, rs16970500, rs5880, rs34748139 | 2 | 0.279 | 2 | 0.847 |
| 36 | GSA.rs11131168, rs28670049, rs7775474, rs9490546, rs76493570 | 26 | 0.448 | 39 | 0.151 |
| 37 | GSA.rs2513345, rs28368589, GSA.rs1341032, rs61851154, rs4755910 | 41 | 0.334 | 58 | 0.894 |
| 38 | rs12623781, rs7789699, rs1427213, rs11076196 | 19 | 0.892 | 31 | 0.430 |
| 39 | GSA.rs765786, rs533857, GSA.rs873601, rs752092, rs115758894 | 22 | 0.137 | 28 | 0.507 |
| 40 | rs2077753, rs13131918, rs737522 | 271 | 0.834 | 427 | 0.436 |
| 41 | rs7531555, rs10800790, rs6716743, rs1120653, GSA.rs1768885 | 5 | 0.481 | 9 | 0.774 |
| 43 | GSA.rs11131168, rs28670049, rs7775474, rs9490546, rs76491632 | 24 | 0.571 | 36 | 0.116 |
| 44 | rs2038486, GSA.rs116450859, rs12913328, rs12151280, rs13053354 | 5 | 0.095 | 5 | 0.674 |
| 45 | rs4657153, rs1498606, rs4715000, rs1925546, rs6082699 | 6 | 0.686 | 8 | 0.950 |
| 46^a^ | rs7593130, rs9864288, rs4554825, rs9788816, 17:48719848 | 27 | 0.739 | 38 | 0.832 |
| 47 | rs2003780, rs11249632, rs11255643, rs2148073, rs12922740 | 9 | 0.729 | 16 | 0.971 |
| 48 | GSA.rs6777173, rs4739119, GSA.rs749377, rs2915409, rs674155 | 4 | 0.561 | 5 | 0.876 |
| 49 | rs1836767, GSA.rs116450859, rs7789699, rs1427213, rs11076196 | 4 | 0.799 | 8 | 0.054 |
| 50 | GSA.rs1260773, rs9497606, rs1177952, rs115758894, rs8091293 | 16 | 0.691 | 24 | 0.101 |
| 51^a^ | rs4322881, rs1039938, GSA.rs72715247, rs8044861, rs7228894 | 47 | 0.287 | 72 | 0.705 |
| 52 | rs401758, rs4921028, rs9296672, GSA.rs1109968, rs1925546 | 7 | 0.721 | 9 | 0.832 |
| 53 | rs401758, rs4921028, rs9296672, GSA.rs58438895, rs1925546 | 7 | 0.721 | 9 | 0.832 |
| 54 | GSA.rs10121074, rs7148304, rs12151280, GSA.rs2015901 | 17 | 0.345 | 28 | 0.985 |
| 55 | rs11695861, rs6970262, rs2374315, GSA.rs4743997, rs60172187 | 13 | 0.330 | 19 | 0.761 |
| 56 | rs2272803, rs12408203, GSA.rs1461338, rs2016461 | 15 | 0.256 | 21 | 0.835 |
| 57 | rs56326015, GSA.rs765786, rs533857, GSA.rs873601, rs752092 | 12 | 0.359 | 14 | 0.423 |
| 58 | rs1110350, GSA.rs1332865, rs4843458, rs12450045, rs1401795 | 54 | 0.286 | 81 | 0.559 |
| 59 | rs342420, rs61401108, rs115758894, rs2337969, GSA.rs6010063 | 8 | 0.453 | 11 | 0.154 |
| 60 | rs2010963, GSA.rs2496495, rs115758894 | 82 | 0.939 | 116 | 0.124 |
| 61 | rs3729547, rs16880691, GSA.rs75038170, rs10405246, rs11672440 | 8 | 0.140 | 9 | 0.283 |
| 62 | rs3729547, rs16880691, GSA.rs75038170, rs10421768, rs11672440 | 8 | 0.140 | 9 | 0.283 |
| 63 | rs342096, rs75681130, rs9347553, rs17106075, GSA.rs17755657 | 8 | 0.069 | 10 | 0.514 |
| 64 | rs7789699, GSA.rs10121074, rs1427213, rs11076196 | 26 | 0.528 | 41 | 0.668 |
| 65 | GSA.rs2073925, GSA.rs4910484, rs1191543, rs12882037, rs8038697 | 11 | 0.074 | 13 | 0.728 |
| 66 | rs6889348, GSA.rs7817160, rs12225758, rs1558508, rs17101923 | 4 | 0.063 | 5 | 0.584 |
| 67 | GSA.rs58438895, rs2814338, rs2357401, rs28377784, rs729533 | 1 | 0.742 | 2 | 0.061 |
| 68 | rs4309752, GSA.rs58438895, rs10766134, rs2239068, rs1403057 | 18 | 0.847 | 24 | 0.378 |
| 69 | GSA.rs9469341, rs1177952, rs17106075, rs2027670, rs8091293 | 9 | 0.695 | 15 | 0.656 |
| 70 | rs13034507, rs11681751, rs6897267, rs10842964, rs60172187 | 4 | 0.410 | 5 | 0.817 |
| 71 | GSA.rs116450859, rs7789699, rs16925478, rs1427213, rs11076196 | 2 | 0.294 | 7 | 0.798 |
| 72 | rs1261411, rs3813477, GSA.rs2073925, rs327034, rs1858993 | 5 | 0.510 | 7 | 0.699 |
| 73 | rs1261411, rs3813477, GSA.rs2073925, rs327025, rs1858993 | 5 | 0.510 | 7 | 0.699 |
| 74 | rs12534437, GSA.rs1811338, GSA.rs35136666, rs487908, rs5758036 | 4 | 0.347 | 9 | 0.155 |
| 75 | GSA.rs765786, rs533857, GSA.rs873601, rs752092, rs5880 | 12 | 0.579 | 18 | 0.814 |
| 76 | rs3729547, GSA.rs116450859, rs4625, GSA.rs1714821, rs73150887 | 10 | 0.091 | 15 | 0.186 |
| 77 | GSA.rs6799023, rs6901613, GSA.rs2709803, rs4986790 | 22 | 0.318 | 29 | 0.803 |
| 79 | rs6716743, rs12492247, rs13096228, GSA.rs58184535, rs6010882 | 3 | 0.233 | 5 | 0.258 |
| 80 | rs6716743, rs12492247, rs13096228, GSA.rs67518897, rs6010882 | 3 | 0.233 | 5 | 0.258 |
| 81 | rs66579885, rs1409961, rs6575815, rs8095535, rs7508763 | 8 | 0.472 | 9 | 0.283 |
| 82 | rs2272803, rs12698097, GSA.rs1361028, rs17272288 | 5 | 0.714 | 7 | 0.355 |
| 83 | rs2385273, rs1902819, GSA.rs2151418, rs11253227, rs12624956 | 3 | 0.540 | 5 | 0.138 |
| 84 | GSA.rs1936980, rs1902819, GSA.rs2151418, rs11253227, rs12624956 | 13 | 0.994 | 20 | 0.968 |
| 85 | GSA.rs765786, rs533857, GSA.rs58438895, GSA.rs873601, rs752092 | 14 | 0.815 | 16 | 0.858 |
| 86 | rs9383658, rs13249564, rs1481687, rs646410 | 10 | 0.096 | 14 | 0.374 |
| 87 | GSA.rs315051, rs12497518, rs77626328, rs7823625, GSA.rs72715247 | 8 | 0.464 | 11 | 0.857 |
| 88 | rs9542920, rs177387, rs2572164, rs5880, rs4814851 | 10 | 0.908 | 20 | 0.636 |
| 89 | rs55717234, GSA.rs10796175, rs2761884, rs115758894 | 53 | 0.450 | 74 | 0.642 |
| 90 | rs2178740, rs11897873, rs202064888, GSA.rs1109968, rs11079309 | 16 | 0.563 | 21 | 0.327 |
| 91 | rs2178740, rs11897873, rs202064888, GSA.rs58438895, rs11079309 | 16 | 0.563 | 21 | 0.327 |
| 92 | rs4760420, rs589258, rs209462, rs56697205 | 38 | 0.077 | 59 | 0.640 |
| 93 | rs10800064, GSA.rs13061104, GSA.rs112932700, GSA.rs7971281, rs16970500 | 6 | 0.276 | 8 | 0.502 |
| 94 | rs2244632, rs7539947, rs13310795, GSA.rs72715247, rs10848660 | 6 | 0.279 | 8 | 0.541 |
| 95 | rs706439, rs11736072, rs583453, GSA.rs72715247, rs35187613 | 2 | 0.266 | 2 | 0.162 |
| 97 | rs2794828, rs62186762, rs909017, rs11568817, rs12600608 | 12 | 0.815 | 15 | 0.186 |
| 98 | rs2794828, rs62186762, rs909017, rs9361235, rs12600608 | 12 | 0.815 | 15 | 0.186 |
| 99 | GSA.rs1532164, rs11756157, rs200041397, rs2237562, rs1549759 | 4 | 0.804 | 6 | 0.553 |
| 100 | rs12989855, rs1531136, rs7754832, rs13273088, rs2915409 | 4 | 0.230 | 4 | 0.798 |
| 101 | rs6834709, rs6947745, rs7006666, rs4554825, rs750456 | 13 | 0.682 | 15 | 0.827 |
| 102 | GSA.rs116450859, rs7641634, rs116122098, rs835760, rs72728737 | 6 | 0.849 | 8 | 0.412 |
| 103 | rs156168, GSA.rs12001512, rs589258, rs8076171 | 48 | 0.312 | 67 | 0.314 |
| 104 | rs11695861, rs6970262, rs2374315, GSA.rs4743997, rs17106075 | 19 | 0.882 | 31 | 0.533 |
| 105 | rs2180419, GSA.rs10503490, rs11113367, GSA.rs75038170, rs55846612 | 4 | 0.623 | 7 | 0.765 |
| 106 | GSA.rs765620, rs7715703, rs115758894 | 122 | 0.491 | 171 | 0.454 |
| 107 | rs7593130, rs4364244, rs1820075, rs2031658, rs8131126 | 23 | 0.795 | 35 | 0.495 |
| 108 | rs4765687, rs11059915, GSA.rs75038170, rs1333416, rs9676118 | 2 | 0.186 | 2 | 0.228 |
| 109 | GSA.rs7681936, rs4554825, rs3741260, rs10765692, rs4613146 | 9 | 0.963 | 10 | 0.369 |
| 111 | rs12701653, rs17106075, rs7320516, rs11072524, GSA.rs72800214 | 0 | 1.000 | 0 | 1.000 |
| 112 | rs4592842, rs10935496, GSA.rs1532164, rs17098351, rs6142656 | 0 | 1.000 | 0 | 1.000 |
| 113 | rs7531501, rs7148304, rs12454570, rs12151280, GSA.rs2015901 | 0 | 1.000 | 0 | 1.000 |
| 114 | rs7531501, rs7148304, rs12454570, rs12151280, rs4381836 | 0 | 1.000 | 0 | 1.000 |
| 115 | rs201492944, rs4529355, GSA.rs76996768, rs948194, rs17106075 | 0 | 1.000 | 0 | 1.000 |
| 116 | rs17075031, rs4529355, GSA.rs76996768, rs948194, rs17106075 | 0 | 1.000 | 0 | 1.000 |
| 117 | GSA.rs2017143, rs847622, rs4896307, rs1485534, rs7950336 | 0 | 1.000 | 0 | 1.000 |
| 118 | GSA.rs2017143, rs847622, rs4896307, rs7950336, GSA.rs35136666 | 0 | 1.000 | 0 | 1.000 |

^a^at least one SNP included in the original definition of the disease signature missing; ^b^dichotomized along the median (3.67) of the BRS score; ^c^dichotomized along the number of acute COVID-19 symptoms (<4 or ≥4) self-reported as either severe or life-threatening

**Supplementary Table 5. Significance (-log[p]) of genotype-phenotype associations of top 100 GWAS SNPs before and after individual adjustment for additional covariates.**

|  | **-log[p] (mean (SD))** | | |
| --- | --- | --- | --- |
| **Covariate** | **PCS** | **PCS-S** | **PCS-R** |
| None | 4.912 (0.302) | 5.290 (0.897) | 4.865 (0.322) |
| Smoking status | 4.161 (0.852) | 4.830 (1.001) | 3.298 (0.822) |
| Pre-existing COPD | 4.430 (0.936) | 4.863 (0.991) | 3.285 (0.772) |
| Pre-existing asthma | 4.225 (0.801) | 4.793 (1.020) | 3.153 (0.772) |
| Pre-existing depression | 4.391 (0.949) | 4.659 (0.877) | 3.210 (0.742) |
| Pre-existing anxiety disorder | 4.409 (0.957) | 4.737 (0.942) | 3.278 (0.723) |
| Pre-existing diabetes mellitus | 4.391 (0.924) | 4.737 (1.004) | 3.323 (0.746) |
| BMI | 3.319 (0.971) | 2.927 (0.902) | 2.103 (0.751) |

COPD: chronic obstructive pulmonary disease; BMI: body mass index.

**Supplementary Table 6. Genotype-phenotype associations of GWAS lead SNPs before and after adjustment for additional covariates.**

| **Score** | **Lead SNP** | **Chromosome** | **Position** | **HWE**  **-log(p)^a^** | **OR (95%CI); -log[p])** |  |
| --- | --- | --- | --- | --- | --- | --- |
|  |  |  |  |  | **Before** | **After** |
| PCS | rs10893121 | 11 | 123854744 | 0.207 | 1.492 (1.263,1.763); 5.603 | 1.664 (1.291,2.144); 4.073 |
|  | rs369729556 | 1 | 88810313 | 1.703 | 0.258 (0.146,0.455); 5.521 | 0.160 (0.060,0.426); 3.624 |
|  | rs853389 | 6 | 14190765 | 1.027 | 1.384 (1.205,1.589); 5.410 | 1.244 (1.022,1.515); 1.528 |
|  | rs149480527 | 11 | 27653122 | 0.069 | 0.487 (0.358,0.663); 5.312 | 0.420 (0.263,0.671); 3.543 |
|  | rs61739314 | 20 | 39990377 | 0.310 | 2.473 (1.671,3.662); 5.214 | 2.226 (1.199,4.133); 1.950 |
|  | rs62459023 | 7 | 79126126 | 0.443 | 0.327 (0.200,0.534); 5.120 | 0.345 (0.176,0.676); 2.718 |
|  | rs2172284 | 12 | 99581888 | 1.249 | 1.389 (1.203,1.607); 5.085 | 1.618 (1.305,2.007); 4.926 |
|  | rs117404345 | 10 | 97656514 | 0.081 | 0.479 (0.347,0.663); 5.067 | 0.497 (0.317,0.779); 2.640 |
|  | rs1569245 | 7 | 9340431 | 1.132 | 1.354 (1.185,1.547); 5.064 | 1.190 (0.982,1.442); 1.115 |
| PCS-S | rs9792535 | 9 | 127166653 | 0.288 | 2.964 (1.998,4.397); 7.181 | 2.780 (1.553,4.977); 3.236 |
|  | rs260913 | 1 | 163968414 | 0.084 | 0.707 (0.618,0.810); 6.231 | 0.685 (0.563,0.834); 3.777 |
|  | rs4600898 | 4 | 150836842 | 0.454 | 0.542 (0.425,0.693); 6.030 | 0.465 (0.268,0.808); 2.178 |
|  | GSA-rs55761436 | 1 | 208998704 | 0.000 | 0.217 (0.114,0.414); 5.449 | 0.152 (0.055,0.418); 3.575 |
|  | rs58659000 | 2 | 43143203 | 0.104 | 2.025 (1.494,2.745); 5.263 | 1.097 (0.579,2.076); 0.110 |
|  | rs12655442 | 5 | 65873081 | 0.521 | 0.735 (0.643,0.840); 5.220 | 0.691 (0.569,0.839); 3.725 |
|  | rs17766082 | 12 | 66046302 | 0.256 | 3.391 (1.985,5.792); 5.107 | 2.848 (1.276,6.359); 1.974 |
|  | rs10465914 | 1 | 16654674 | 0.915 | 1.357(1.186,1.553); 5.048 | 1.408 (1.157,1.714); 3.204 |

| PCS-R | rs73562426 | 8 | 17428762 | 0.840 | 0.298 (0.182,0.486); 5.888 | 0.290 (0.139,0.604); 3.018 |
| --- | --- | --- | --- | --- | --- | --- |
|  | rs56098254 | 17 | 12659294 | 0.350 | 0.711 (0.617,0.819); 5.621 | 0.666 (0.538,0.824); 3.738 |
|  | rs1922263 | 12 | 104871892 | 1.253 | 0.381 (0.254,0.570); 5.543 | 0.553 (0.233,1.314); 0.746 |
|  | rs17058997 | 6 | 130755711 | 0.035 | 1.591 (1.305,1.940); 5.370 | 1.554 (1.162,2.079); 2.526 |
|  | rs6808586 | 3 | 85168434 | 1.033 | 1.354 (1.183,1.532); 5.346 | 1.282 (1.059,1.551); 1.961 |
|  | GSA-rs34481469 | 11 | 114684696 | 0.000 | 2.955 (1.843,4.738);5.160 | 2.618 (1.312,5.222); 2.199 |
|  | rs369729556 | 1 | 88810313 | 1.703 | 0.282 (0.163,0.490); 5.155 | 0.170 (0.065,0.444); 3.528 |
|  | rs6754502 | 2 | 147576544 | 1.818 | 1.781(1.381,2.297); 5.052 | 1.384 (0.940,2.037); 1.001 |

HWE: Hardy-Weinberg equilibrium; OR: allelic odds ratio estimated for the minor SNP allele; 95%CI: 95% confidence limit of OR. Additional covariates considered for adjustment included smoking, pre-existing depression, pre-existing anxiety disorder, pre-existing asthma, pre-existing COPD, pre-existing diabetes mellitus and BMI. ^a^For quality control, a -log(p)>5 criterion was used to exclude SNPs that were not in HWE. All lead SNPs clearly fell far short of this criterion.
